# Predicting Impact Loads on Polymer Materials from Laser-Induced Cavitation Bubble Collapse Using Random Forest Regression

**DOI:** 10.3390/mi17091083

**Published:** 2026-09-15

**Authors:** Muhammad Farkhan Abdillah, Kazuaki Inaba

**Affiliations:** Department of Transdisciplinary Science and Engineering, Institute of Science Tokyo, Tokyo 152-8550, Japan; abdillah.m.0228@m.isct.ac.jp

**Keywords:** cavitation, polymers, machine learning, PVDF sensor, laser-induced bubble collapse, high-speed imaging

## Abstract

Cavitation bubble collapse generates impact loads that can initiate surface damage and accelerate material degradation in hydraulic and fluid-handling systems. Predicting these loads remains challenging because the collapse response depends on nonlinear interactions among bubble dynamics, stand-off distance, and polymer mechanical, acoustic, and viscoelastic properties. This study developed machine-learning regression models to predict impact loads from laser-induced single-bubble collapse on polyethylene, polytetrafluoroethylene (PTFE), polyamide, and antistatic polyethylene terephthalate (antistatic PET). Experiments were performed using a pulsed Nd:YAG laser at energies of 12.5, 25, and 50 mJ and normalized stand-off distances of γ = 1–5, producing 180 measurements. Impact loads were measured using a force-calibrated PVDF sensor with material-specific calibration equations. Bubble radius was obtained from high-speed imaging, whereas collapse time was determined from the PVDF voltage-time response. Thirteen input variables were used to train linear, ridge, LASSO, multilayer perceptron, and random forest regressors with Bayesian optimization and grouped five-fold cross-validation. Random forest regression achieved the best performance, with RMSE=0.6820N, MAE=0.5339N, $R^{2}=0.9168$, and MAPE=12.44%. SHAP analysis identified collapse time, acoustic impedance, and loss modulus as dominant predictors. The model is therefore suitable for trend prediction within the tested experimental domain.

## 1. Introduction

Cavitation damage is a major factor limiting the reliability, efficiency, and service life of hydraulic machinery, including pumps, hydro turbines, marine propellers, and fluid transport systems [1]. It begins when the local pressure falls below the liquid vapor pressure, producing vapor-filled cavities that are transported into higher-pressure regions and collapse violently [2]. In practical systems, cavitation generally occurs as clouds of interacting bubbles rather than isolated cavities. High-speed observations have shown that the strongest hydrodynamic loads arise during the final stage of cloud collapse, when collective energy focusing produces substantially greater impact intensities than individual bubble collapses [3].

The erosive intensity of a cavitation cloud depends strongly on its morphology. Fragmented and irregular clouds often produce greater damage because they break into smaller clusters and filaments that concentrate impact energy over localized surface regions [4]. During collapse, the surrounding liquid accelerates toward the cavity center, generating high-velocity micro-jets and shock waves that may produce extremely high local pressures [5]. Repeated exposure to these pressure pulses causes progressive surface deformation and cavitation erosion.

At the microscopic level, erosion develops through the cumulative action of repeated impacts. Deep pits commonly result from successive cavitation events occurring at the same location rather than from a single collapse [6]. The process typically begins with plastic deformation and work hardening, followed by stress accumulation, crack initiation, and crater formation once the material fatigue limit is exceeded [7]. Continued erosion alters the geometry of impellers, turbine runners, and other hydraulic components, leading to increased vibration and noise, reduced hydraulic performance, and premature failure of bearings and seals [1]. These effects increase maintenance requirements, operational downtime, and overall lifecycle costs.

Despite its practical importance, cavitation cloud erosion remains difficult to study because of the stochastic interactions among large numbers of bubbles. Single-bubble cavitation experiments therefore provide a controlled and reproducible approach for examining collapse dynamics, impact-load generation, and material response. By removing the interactions present in cavitation clouds, these experiments allow the governing mechanisms to be studied under well-defined conditions [8].

Single bubbles are commonly generated by focused pulsed lasers, including Q-switched Nd:YAG, ruby, and Holmium:YAG systems, or by electrical spark discharge [9,10,11,12]. These methods provide good spatial and temporal repeatability and enable precise control of the dimensionless stand-off distance between the bubble inception point and the solid boundary. Previous studies have demonstrated that the collapse of a single laser-induced bubble near a solid boundary can reproduce the fundamental loading mechanisms associated with cavitation erosion, particularly shock-wave emission and microjet impact. These transient mechanisms can induce local plastic deformation and contribute to pit initiation [10,13]. Thus, the present model should be interpreted as predicting the local impulsive load generated by an individual collapse event, rather than directly predicting cumulative erosion or fatigue damage under pump cavitation conditions. Near a boundary, bubble collapse becomes asymmetric and produces a high-velocity liquid micro-jet together with intense shock waves during the final collapse stage [8,14].

Because these processes occur over nanosecond-to-microsecond timescales, advanced diagnostic methods are required. Bubble dynamics are commonly recorded using ultra-high-speed imaging combined with shadowgraphy or schlieren techniques to visualize interface motion and shock-wave propagation [15]. Particle image velocimetry may be used to quantify the surrounding flow field, while miniature hydrophones, optical-fiber probes, and PVDF piezoelectric sensors are employed to measure impulsive pressure loads [15]. Impacts are often directed onto well-characterized materials, including aluminum, indium, structural alloys, polymers, and gelatin-based media [8,10,14]. Subsequent analyses using optical profilometry, scanning electron microscopy, and micro-indentation provide quantitative information on pit geometry, work hardening, and crack initiation. Such experimental datasets are essential for validating numerical simulations and developing predictive models of cavitation-induced loads and erosion.

Although cavitation in pumps usually occurs as repeated multiple-bubble or cloud-bubble collapse rather than isolated single-bubble collapse, the present study intentionally focuses on a single Nd:YAG laser-induced bubble as a controlled elementary collapse event. This approach allows the collapse load generated by one bubble to be evaluated under different material properties, laser pulse energies, and stand-off distances, while excluding bubble–bubble interaction, shock-wave-induced secondary collapse, and the complex pressure-wave propagation associated with cloud cavitation. Previous studies have shown that single laser-induced bubble collapse near a solid wall can generate the fundamental loading mechanisms of cavitation erosion, including shock-wave emission, microjet impact, local plastic deformation, and pit initiation [10,13]. Therefore, the present model should be interpreted as a prediction of local impulsive load caused by an individual collapse event, rather than as a direct prediction of cumulative erosion or fatigue damage in pump cavitation.

Machine-learning methods have recently been applied to cavitation problems in several engineering fields. One-dimensional convolutional autoencoders have been used to reconstruct and predict cavitation pressure signals around underwater moving bodies, including cases with limited experimental data [16]. In acoustic cavitation, supervised models based on theoretical bubble-dynamics equations have classified stable and transient regimes from physical and acoustic parameters [17]. Algorithms such as k-nearest neighbors and support vector machines have also been used to estimate shock-induced cavitation levels as a function of fluid temperature in biomedical applications [18]. In addition, hybrid random-forest and back-propagation neural-network models have been developed to predict bubble-collapse strength under variations in viscosity, surface tension, and other physical conditions [19]. These studies demonstrate the potential of data-driven methods for cavitation-related prediction. However, relatively limited attention has been given to quantitatively linking controlled single-bubble impact measurements with the dynamic mechanical and viscoelastic properties of polymer targets. In particular, how bubble dynamics, acoustic coupling, and polymer viscoelastic response jointly contribute to the measured impact load under different stand-off conditions remains insufficiently understood.

The reliability of machine-learning models depends strongly on the selection of training hyperparameters. Hyperparameter optimization aims to determine appropriate values for model architecture, regularization, learning rate, batch size, and training duration [20]. Poorly selected settings may result in underfitting, overfitting, or inefficient computation, whereas systematic optimization improves predictive accuracy, generalization, and reproducibility. Bayesian optimization is particularly suitable for computationally expensive models because it uses a surrogate representation of the validation response to identify promising configurations with relatively few evaluations [21]. Integrating hyperparameter optimization into cavitation impact-load prediction is therefore important for developing models that are accurate, computationally practical, and robust for engineering applications.

In this study, a regression framework was developed to predict the impact loads generated by laser-induced single-bubble collapse on polymer materials. Impact loads were measured experimentally using a force-calibrated polyvinylidene fluoride (PVDF) sensor for four representative polymers: polyethylene, PTFE, polyamide, and antistatic PET. The experiments were conducted at different normalized stand-off distances using bubbles generated by a pulsed Nd:YAG laser. Thirteen input variables, including material properties, bubble-dynamics parameters, experimental conditions, acoustic characteristics, and viscoelastic properties, were used to construct the predictive models. The liquid properties were not included as model inputs because all experiments were conducted in water under the same room-temperature and atmospheric-pressure conditions. Several regression algorithms were evaluated, including linear regression, ridge regression, LASSO regression, random forest regression, and multilayer perceptron regression. Model performance was assessed using standard regression metrics, and feature-importance analysis was performed to identify the variables that contributed most strongly to impact-load prediction. Particular attention was given to the relative roles of bubble dynamics, acoustic coupling, and the frequency-dependent viscoelastic response of the polymer, as well as to how their contributions vary with the stand-off condition. The proposed framework therefore provides both a data-driven method for predicting single-bubble impact loads and a means of examining how bubble and material characteristics jointly influence the transient loading of polymer surfaces.

## 2. Materials and Methods

### 2.1. PVDF Sensor Calibration

The polyvinylidene fluoride (PVDF) sensor (DT1-052K, Measurement Specialties, Inc., Norristown, PA, USA) was used to measure the impact load generated by the collapse of a laser-induced bubble on solid materials. In this study, the tested materials consisted of polyethylene, PTFE, polyamide, and antistatic PET. Before conducting the bubble collapse experiments, the PVDF sensor was calibrated to establish the relationship between the measured voltage output and the corresponding impact force. The high-speed camera was used to capture the coefficient of restitution for each material. The calibration procedure was conducted using a ball-drop impact method similar to that reported in [22]. The coefficient of restitution for each material was calculated from the initial drop height and the rebound height of the ball after impact. Because the bubble collapse measurements were performed under submerged conditions, the ball-drop calibration experiments were also conducted with the specimens submerged in water. The top surface of each specimen was positioned 5 mm below the water surface to replicate the conditions of the bubble collapse experiments.

In the calibration test, a ball with a known mass was released from prescribed drop heights onto the PVDF sensor. The impact of the falling ball generated a transient voltage signal. Because the contact between the ball and the sensor occurred within a short duration, the mean impact force during the first peak of the signal was estimated using the impulse–momentum relationship as

(1) $$F_{\mathrm{mean}}=\frac{1}{\Delta t}\int_{t_{s}}^{t_{e}}F\left(t\right)\;dt=\frac{m}{\Delta t}\left(v_{1}+v_{2}\right),$$

where $F_{\mathrm{mean}}$ is the mean impact force, F(t) is the time-dependent impact force, *m* is the mass of the ball, $v_{1}$ is the velocity of the ball immediately before impact, $v_{2}$ is the rebound velocity after impact, and $\Delta t=t_{e}-t_{s}$ represents the duration of the first impact peak.

The velocities before and after impact are related to the drop and rebound heights by

(2) $$v_{1,2}=\sqrt{2gh_{1,2}},$$

where *g* is the gravitational acceleration, $h_{1}$ is the initial drop height, and $h_{2}$ is the rebound height. By defining the coefficient of restitution as

(3) $$e=\frac{h_{2}}{h_{1}},$$

the mean impact force can be expressed as

(4) $$F_{\mathrm{mean}}=\frac{m}{\Delta t}\sqrt{2gh_{1}}\left(1+\sqrt{e}\right).$$

**Figure 1:**
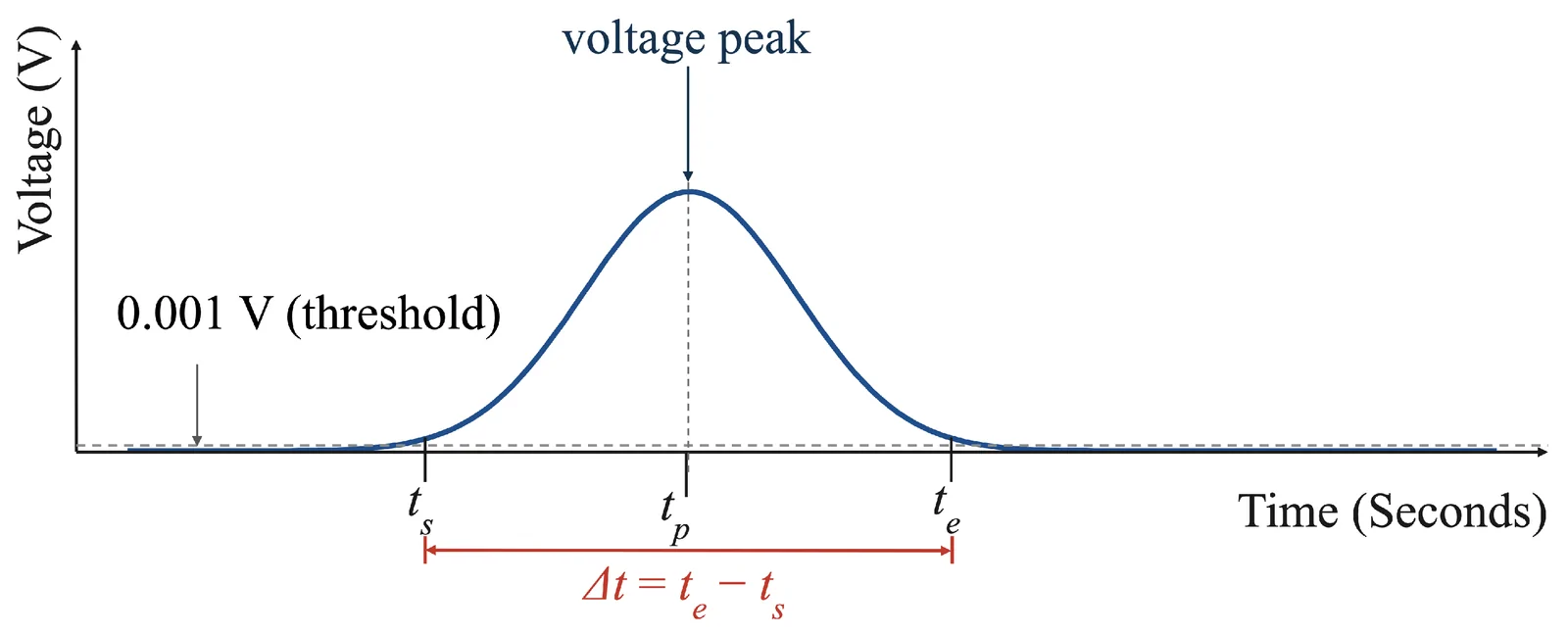
Definition of the first-peak duration from the PVDF voltage response. The start time $t_{s}$ and end time $t_{e}$ correspond to the upward and downward crossings of the 0.001V threshold, respectively, and the peak duration is defined as $\Delta t=t_{e}-t_{s}$.

For each drop height, the voltage response of the PVDF sensor was recorded, and the corresponding mean impact force was calculated using the duration of the first voltage peak. The peak duration was defined as $\Delta t=t_{e}-t_{s}$, where $t_{s}$ is the time at which the voltage signal first exceeds the threshold before reaching the peak, and $t_{e}$ is the time at which the signal falls below the threshold after the peak. These times were automatically identified using a peak-detection program with a voltage threshold of 0.001 V. This threshold was selected after confirming that it was above the baseline noise level of the PVDF signal. The definition of the peak duration is illustrated in Figure 1. The calculated force values were then correlated with the measured peak voltage to obtain the voltage-to-force calibration coefficient. This coefficient was subsequently used to convert the PVDF voltage signals obtained during the bubble collapse experiments into impact load values. According to the Piezo Film Sensors Technical Manual [23], PVDF piezo film exhibits a broadband frequency response; however, the effective frequency response of the measurement system depends on the electrical loading, sensor capacitance, and signal-conditioning circuit. In the present experiments, the same PVDF sensor and amplifier settings were used for both the falling-ball calibration and bubble-collapse measurements, with the amplifier low-cut and high-cut frequencies set to 1 kHz and 100 kHz, respectively. Therefore, both measurements were subjected to the same band-limited signal-conditioning system. Because the falling-ball and bubble-collapse impacts have different characteristic timescales and spectral contents, the reported impact loads should be interpreted as band-limited peak loads measured within the effective bandwidth of the present measurement system. The coefficient of restitution in water ranged from 0.54 to 0.68 [22] depending on the target material. For a 1.00±0.02 g ball, the drop-impact calibration was conducted at initial heights of 3, 6, 9, and 50 cm for PTFE, polyamide, and antistatic PET. The additional 50 cm condition was introduced to extend the experimentally calibrated voltage and force ranges toward the higher bubble-collapse impact loads measured for these materials. For polyethylene, calibration at 3, 6, and 9 cm was sufficient because the measured bubble-collapse impact loads remained within the corresponding calibration range.

The coefficients of restitution for polyethylene, polyamide, and antistatic PET were obtained from the submerged ball-drop experiments reported by Abdillah et al. [22], which were conducted at initial drop heights of 3, 6, and 9 cm. The coefficient of restitution for PTFE was determined in the present study using the same submerged ball-drop procedure. For the additional 50 cm calibration condition, the rebound height could not be measured reliably with the present high-speed imaging setup because the larger rebound motion required a wider field of view. Therefore, the corresponding material-specific coefficients of restitution determined at drop heights of 3–9 cm were used in Equation (4) to calculate the impact force at the 50 cm condition. The uncertainty associated with the coefficient of restitution was included in the calibration-force uncertainty analysis described below.

The resulting voltage and force ranges over which the linear calibration relations were established, together with the calibration equations and coefficients of determination ($R^{2}$), are summarized in Table 1.

The uncertainty in the calibration force calculated using Equation (4) was evaluated by propagating the uncertainties in ball mass, coefficient of restitution, and impact duration. The uncertainty symbols are defined as $u_{m}$, $u_{e}$, and $u_{\Delta t}$, respectively. In this analysis, $u_{m}=0.02\;g$, while $u_{e}$ and $u_{\Delta t}$ were taken from the coefficient-of-restitution and impact-duration measurements, respectively. Assuming independent uncertainty sources, the relative uncertainty was calculated as

$$\frac{u_{F_{\mathrm{mean}}}}{F_{\mathrm{mean}}}=\sqrt{{\left(\frac{u_{m}}{m}\right)}^{2}+{\left(\frac{u_{e}}{2\sqrt{e}\left(1+\sqrt{e}\right)}\right)}^{2}+{\left(\frac{u_{\Delta t}}{\Delta t}\right)}^{2}}.$$

The contribution from the ball-mass uncertainty was approximately 2.0%, that from the coefficient of restitution was approximately 0.36–0.55%, and that from the impact-duration measurement ranged from approximately 1.01% to 5.77%, depending on the material and drop height. The resulting combined relative uncertainty in the calculated calibration force was approximately 2.27–6.13%. Thus, the calibration-force uncertainty was primarily associated with the ball mass and impact-duration measurements, whereas the contribution from the reported uncertainty in the coefficient of restitution was comparatively small [22].

For the additional 50 cm calibration condition, the material-specific coefficient of restitution reported by Abdillah et al. [22] was used because the rebound height could not be measured reliably with the present high-speed imaging setup. Therefore, although the reported uncertainty in the coefficient of restitution was included in the uncertainty propagation, a possible impact-velocity dependence of the coefficient of restitution at 50 cm was not independently quantified.

**Figure 2:**
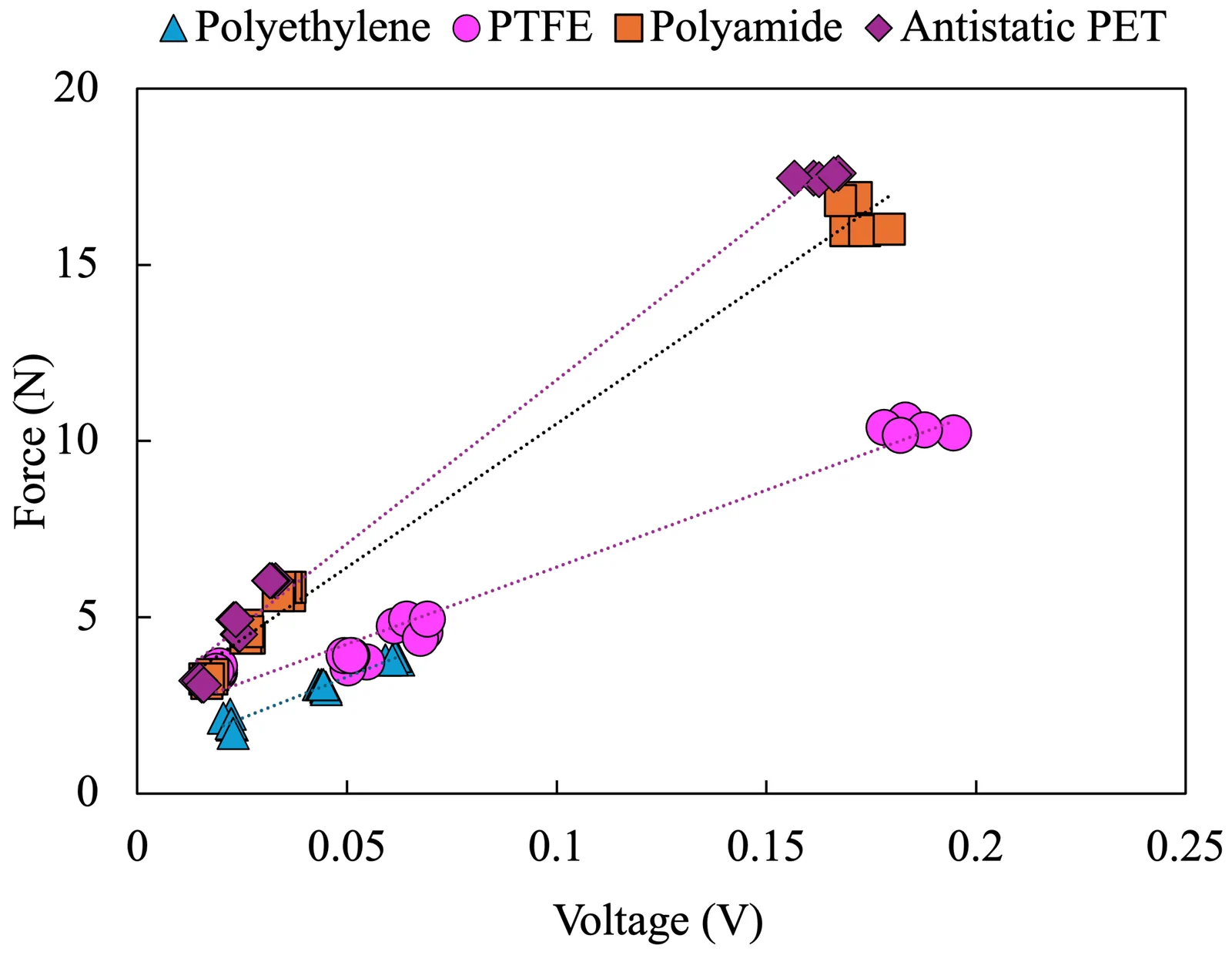
PVDF Force Calibration for All Materials.

The PVDF force-calibration results are shown in Figure 2. The calibration data for polyethylene, PTFE, polyamide, and antistatic PET were obtained following the procedure reported in Ref. [22], while the present study includes PTFE as an additional material for force calibration. For each material, a linear regression line was fitted to the relationship between the measured PVDF voltage and the calculated impact force. The resulting calibration coefficients are summarized in Table 1.

### 2.2. Experimental Setup and Dataset Preparation

**Figure 3:**
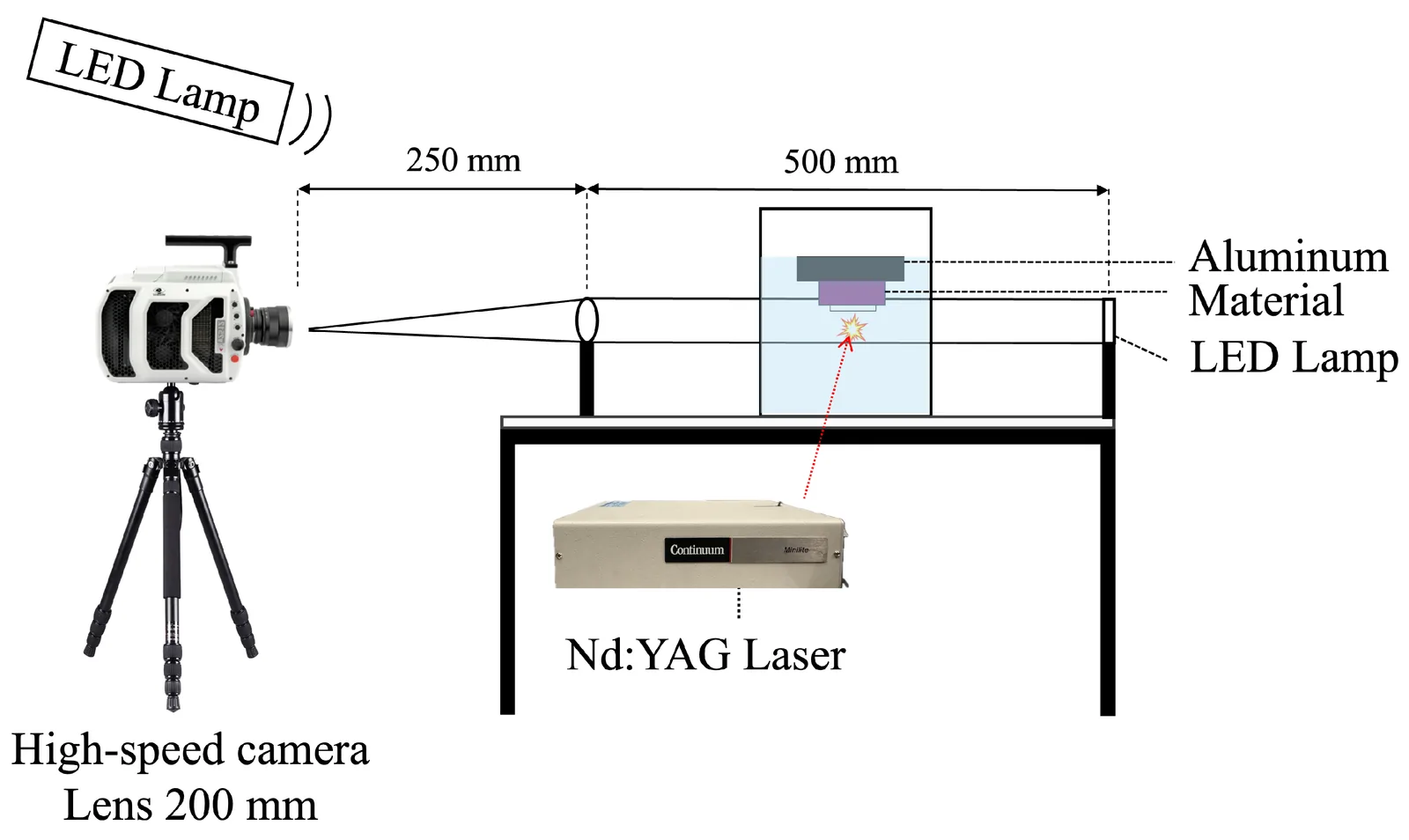
High-speed camera visualization of laser-induced bubble collapse.

The bubble collapse and the generated shock wave occur within a microsecond time scale. Therefore, a reliable high-speed imaging system is required to observe these phenomena. In this experiment, a Phantom high-speed camera was used to capture the bubble dynamics at a high frame rate. As shown in Figure 3, the camera was operated at 110,000 fps, and a backlight LED was used to provide sufficient illumination for capturing the bubble collapse process.

An Nd:YAG laser was employed to generate the cavitation bubble. As illustrated in Figure 3, the laser beam passed through a beam expander and a focusing lens before being focused into the water. The laser wavelength was 1064 nm, and the laser system was capable of producing 50 mJ of maximum energy in a single pulse. The focused laser energy generated a cavitation bubble, which produced a shock wave during the growth and collapse phases.

Bubble-collapse impact measurements for polyethylene, polyamide, and antistatic PET at stand-off distances of γ = 1–5 were previously reported in [22]. The present study extends the previous experiments by including PTFE and additional laser-energy conditions of 12.5 and 25 mJ. To improve the machine learning model, the present study expands the experimental input conditions by adding PTFE as an additional material case and introducing lower laser energy levels of 12.5 mJ and 25 mJ. Therefore, the bubble collapse experiments in this study were conducted using four solid materials, namely polyethylene, PTFE, polyamide, and antistatic PET. The stand-off distance was defined as

(5) $$\gamma =\frac{h}{R_{\max}},$$

where *h* is the distance between the bubble center and the solid surface, and $R_{\max}$ is the maximum bubble radius. The experiments were conducted at five stand-off distances of γ=1,2,3,4, and 5, and three laser energy levels of 12.5 mJ, 25 mJ, and 50 mJ. In total, 60 different experimental conditions were obtained. Each condition was repeated three times, resulting in 180 experimental datasets.

**Figure 4:**
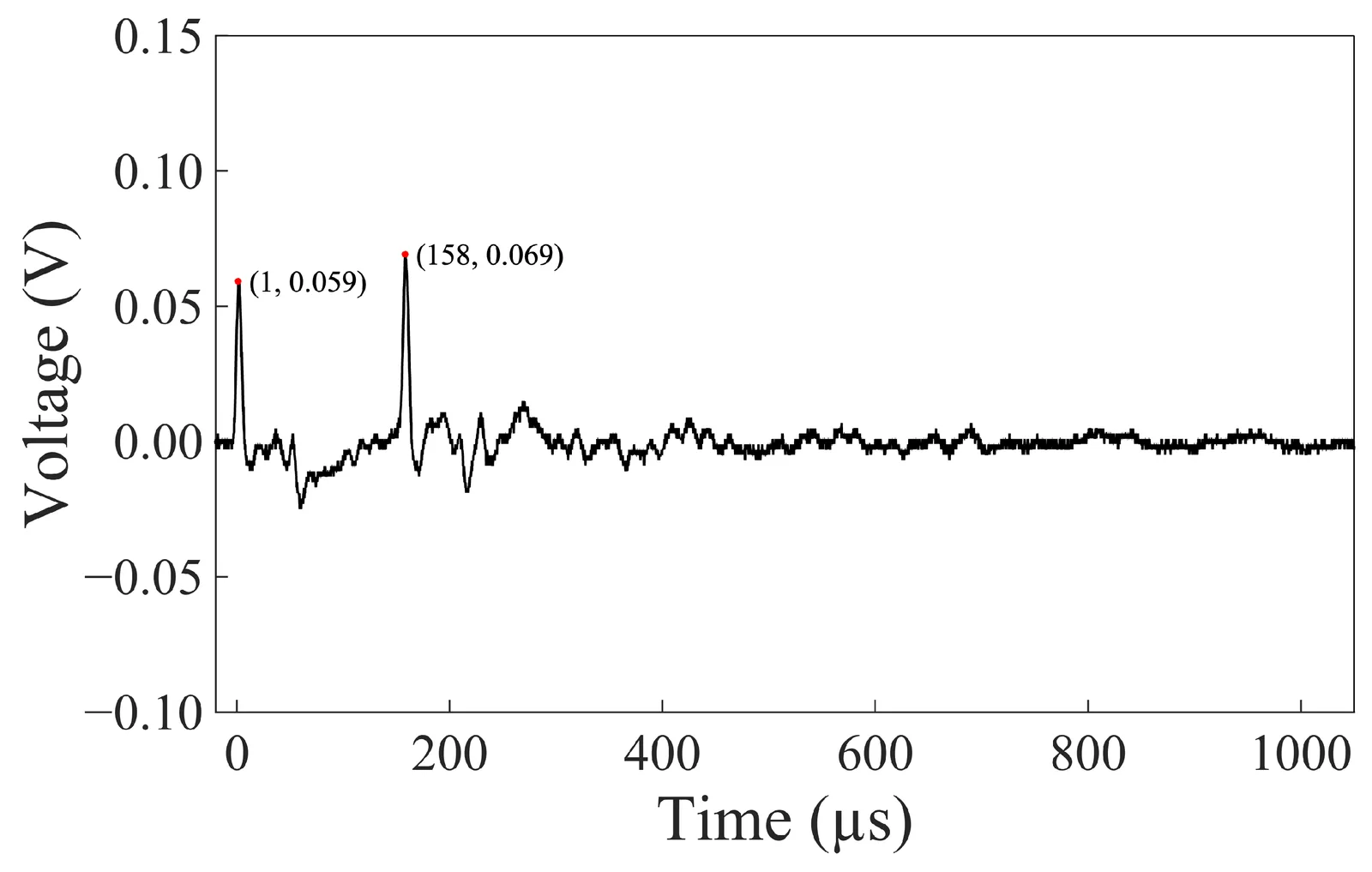
Representative PVDF voltage-time signal for laser-induced bubble collapse on antistatic PET at γ=3 and a laser energy of 25 mJ. The first peak corresponds to the initial laser-induced shock-wave response, and the second peak corresponds to the mechanical impact associated with the first bubble collapse. The collapse time $t_{c}$ is defined as the time interval between these two peaks.

The PVDF sensor was used to measure the impact load generated by the bubble collapse. An example of the impact force received on the antistatic PET surface at 25 mJ laser energy and γ=3 is shown in Figure 4. The measured impact signal can be divided into two main parts. The first peak corresponds to the initial shock wave, which occurs at approximately t=0 μs. The second peak corresponds to the bubble-collapse impact load. Since the bubble collapse time varies depending on the material and stand-off distance, the maximum impact load was extracted for each experimental condition.

**Figure 5:**
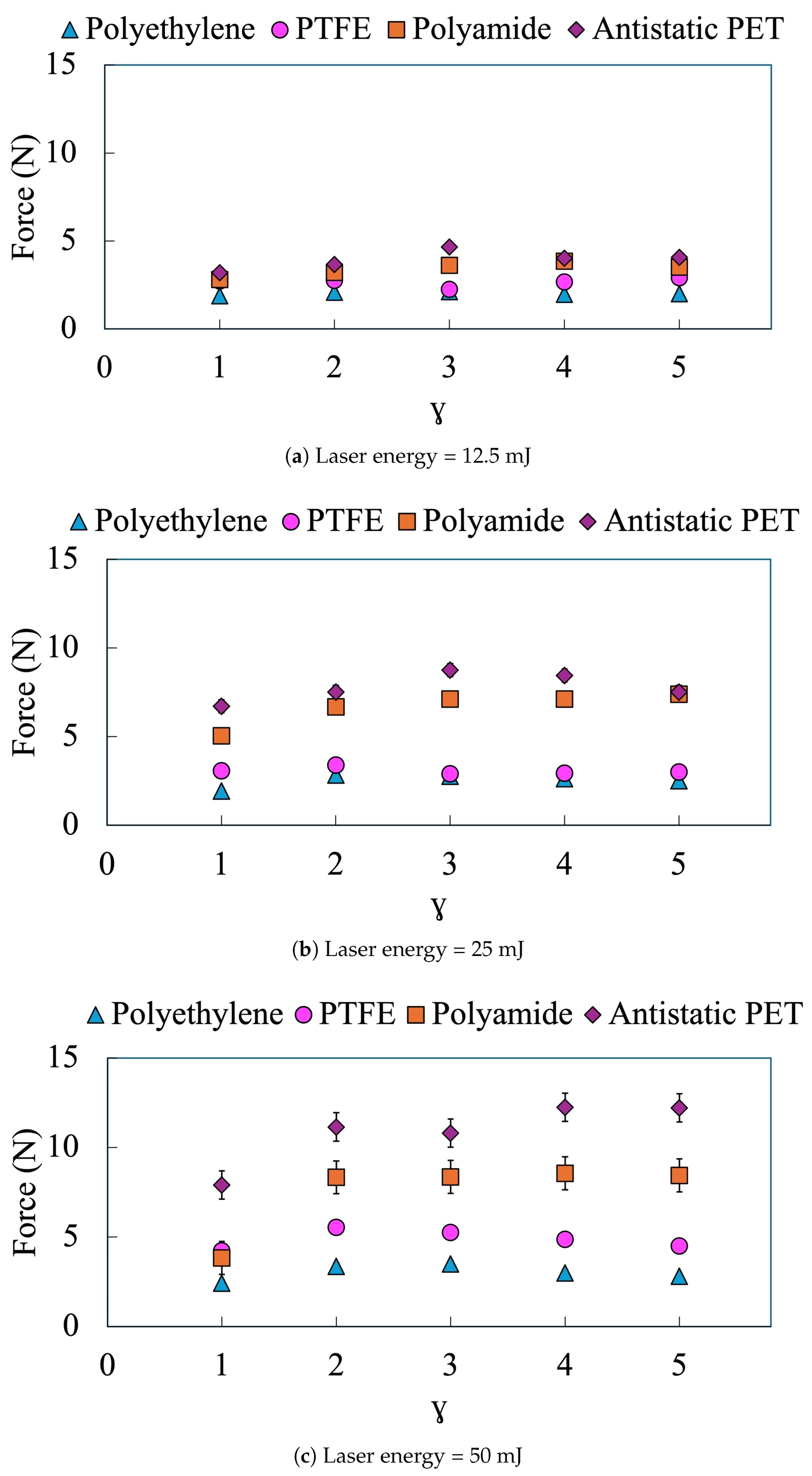
Maximum bubble-collapse impact loads at different stand-off distances for all tested materials under different laser energies: (**a**) 12.5 mJ, (**b**) 25 mJ, and (**c**) 50 mJ.

In this study, the measured PVDF voltage signal was converted into impact load using the calibration coefficients summarized in Table 1. The target output of the machine learning model was the bubble-collapse impact load, defined as the second peak in the PVDF signal. This peak represents the maximum force generated by bubble collapse and occurs approximately 158 μs after laser irradiation. Similarly, the bubble-collapse impact load for different materials, laser energies, and stand-off distances was calculated using the corresponding calibration equation for each material. To improve the reliability of the results, each experimental condition was repeated three times. The maximum bubble-collapse impact loads at different stand-off distances for all tested materials are shown in Figure 5a–c, corresponding to laser energies of 12.5 mJ, 25 mJ, and 50 mJ, respectively. The bubble-collapse impact load was used as the output variable in the machine-learning model, while the input variables consisted of bubble dynamics, stand-off distance, laser energy, acoustic properties, and mechanical and viscoelastic properties of the polymer.

### 2.3. Material Properties

The intrinsic mechanical properties of the investigated polymer materials are summarized in Table 2. Four engineering polymers, namely polyethylene, PTFE, polyamide, and antistatic PET, were selected because they exhibit a broad range of mechanical stiffness and acoustic characteristics. This variation enables a systematic investigation of the influence of material properties on the impact loads generated by laser-induced bubble collapse.

The density, flexural modulus, and tensile yield strength of each polymer were obtained from engineering material databases, whereas the longitudinal wave velocity was collected from published literature [24,25,26,27,28]. Since pressure-wave transmission during bubble collapse depends on the acoustic interaction between the surrounding water and the solid target, the equivalent acoustic impedance at the liquid–solid interface was adopted as one of the machine learning input variables. The equivalent acoustic impedance is expressed as

(6) $$Z=\frac{1}{\left(\frac{1}{z_{1}}\right)+\left(\frac{1}{z_{2}}\right)},$$

where $z_{1}$ and $z_{2}$ denote the intrinsic acoustic impedances of water and the polymer, respectively. The intrinsic acoustic impedance of water is calculated as

(7) $$z_{1}=c_{1}\rho_{1},$$

where $c_{1}$ is the speed of sound in water ($c_{1}=1482$ m/s) and $\rho_{1}$ is the water density ($\rho_{1}=998$ kg/m^3^). Similarly, the intrinsic acoustic impedance of the polymer is determined by

(8) $$z_{2}=c_{2}\rho_{2},$$

where $\rho_{2}$ is the polymer density and $c_{2}$ is the longitudinal wave velocity. To maintain consistency with the available material properties, the longitudinal wave velocity was estimated from the flexural modulus according to

(9) $$c_{2}=\sqrt{\frac{E_{f}}{\rho_{2}}},$$

where $E_{f}$ denotes the flexural modulus of the polymer. The equivalent acoustic impedance obtained from Equation (6) characterizes the acoustic coupling between the liquid and the solid surface and was employed as one of the machine learning input variables. The experiments were conducted in distilled water at room temperature (20 °C) under atmospheric pressure.

### 2.4. Maximum Bubble Radius

The maximum bubble radius was determined from high-speed videos recorded at 110,000 fps. The experiments were conducted using four polymer materials: polyethylene, PTFE, polyamide, and antistatic PET. For each material, the bubble evolution was examined at laser pulse energies of 12.5 mJ, 25 mJ, and 50 mJ and normalized stand-off distances of γ = 1, 2, 3, 4, and 5.

A custom image-processing program developed in Python 3.9.6 was used to detect and measure the laser-induced cavitation bubble in each video frame. First, a region of interest containing the bubble was selected and applied to the image sequence. Each image was converted to grayscale and smoothed using a Gaussian filter to reduce image noise. The bubble was then separated from the background using Otsu thresholding. Morphological opening and closing operations were applied to remove isolated pixels and close small gaps in the bubble boundary. The enclosed bubble region was subsequently filled, and the external contour was identified.

The laser-induced cavitation bubbles observed in the high-speed images were not perfectly spherical and generally exhibited an elongated projected shape. To account for this non-sphericity while estimating the three-dimensional bubble size from the two-dimensional images, the bubble was approximated as an axisymmetric ellipsoid with semi-axes $R_{L}$, $R_{V}$, and $R_{V}$, where $R_{L}$ and $R_{V}$ are the longitudinal and transverse radii obtained from an ellipse fitted to the detected bubble contour.

The equivalent bubble radius, $R_{\mathrm{eq}}$, was defined as the radius of a sphere having the same estimated volume as the axisymmetric ellipsoid. The ellipsoid volume is

$$V_{\mathrm{ellipsoid}}=\frac{4}{3}\pi R_{L}R_{V}^{2},$$

and equating this volume to that of an equivalent sphere,

$$V_{\mathrm{sphere}}=\frac{4}{3}\pi R_{\mathrm{eq}}^{3},$$

gives

(10) $$R_{\mathrm{eq}}={\left(R_{L}R_{V}^{2}\right)}^{1/3},$$

where $R_{L}$ is the radius along the major axis of the fitted ellipse and $R_{V}$ is the radius along the minor axis. The maximum bubble radius, $R_{\max}$, was defined as the largest equivalent radius measured during the bubble expansion stage,

(11) $$R_{\max}=\max\left[R_{\mathrm{eq}}\left(t\right)\right].$$

This volume-equivalent definition was adopted because the subsequent analysis required a characteristic three-dimensional bubble size, particularly for estimating the bubble natural frequency. In contrast, a projected-area-equivalent radius characterizes the two-dimensional image area and does not directly provide an estimate of bubble volume. The present definition therefore preserves the estimated bubble volume under the assumption of axisymmetry.

Three repeated experiments were conducted for each condition. The reported maximum bubble radius is expressed as the mean value together with its standard deviation in Table 3. Across all conditions, the maximum bubble radius showed low absolute variability, with a mean variance of $0.0154\;{\mathrm{mm}}^{2}$ and a root-mean variation of approximately 0.124mm. The relative radius variability, calculated as $\sigma_{R}/R_{\max}$, averaged 15.0% and reached a maximum of 29.1%.

### 2.5. Bubble Collapse Time

The bubble collapse time was determined from the PVDF voltage-time signal using the two characteristic peaks shown in Figure 4. The first peak corresponds to the initial laser-induced shock-wave response, whereas the second peak corresponds to the mechanical impact associated with the first bubble collapse. The collapse time was therefore defined as $t_{c}=t_{\mathrm{collapse}}-t_{\mathrm{initial}}$, where $t_{\mathrm{initial}}$ and $t_{\mathrm{collapse}}$ are the times of the first and second PVDF peaks, respectively.

The PVDF signal was used as the primary reference for determining the collapse time because it provides a finer temporal representation of the collapse-associated impact than identification of the collapse frame from the high-speed images. The camera was operated at 110,000fps, corresponding to a temporal interval of approximately 9.09 μs between successive frames. The high-speed image sequences were therefore used as supporting observations to confirm the bubble-growth and collapse sequence and to determine the maximum bubble radius.

The collapse time was measured for polyethylene, PTFE, polyamide, and antistatic PET at laser pulse energies of 12.5, 25, and 50 mJ and normalized stand-off distances of γ = 1–5. Three repeated measurements were conducted for each condition, and the resulting collapse times are reported as mean ± standard deviation in Table 4.

### 2.6. Frequency-Dependent Viscoelastic Properties

The mechanical response of a polymer depends on the frequency of the applied deformation. To evaluate the viscoelastic properties under a loading rate representative of bubble collapse, the natural frequency of the laser-induced bubble was estimated using the Minnaert equation:

(12) $$f_{M}=\frac{1}{2\pi R_{\max}}\sqrt{\frac{3\gamma_{\mathrm{gas}}P_{0}}{\rho_{w}}},$$

where $R_{\max}$ is the maximum bubble radius, $\gamma_{\mathrm{gas}}$ is the ratio of specific heats of the gas, $P_{0}$ is the ambient absolute pressure, and $\rho_{w}$ is the density of the surrounding water. In the present calculation, $\gamma_{\mathrm{gas}}=1.4$, $P_{0}=101,325\;\mathrm{Pa}$, and $\rho_{w}=997\;\mathrm{kg}\;m^{-3}$. The value of $R_{\max}$ must be expressed in meters to obtain $f_{M}$ in hertz. Since the Minnaert frequency is inversely proportional to the bubble radius, this radius variability corresponds directly to an average estimated frequency variability of approximately 15.0%, with a maximum variation of approximately 29.1%. Since the maximum bubble size depends on the energy deposited by the laser pulse, fluctuations in the laser output can lead to variations in the measured bubble axes, the estimated bubble volume, and consequently the equivalent radius defined in Equation (10).

The calculated bubble natural frequency was adopted as the representative loading frequency for determining the frequency-dependent viscoelastic properties of each polymer:

(13) $$f=f_{M},$$

where *f* denotes the frequency used to evaluate the storage and loss moduli. Although bubble collapse produces a transient load with a broad frequency spectrum, the Minnaert frequency provides a physically meaningful characteristic timescale associated with the radial oscillation of the bubble. It was therefore used as an approximate loading frequency.

To evaluate the consistency of the characteristic timescale obtained from this approximation, the Minnaert duration was calculated as the inverse of the Minnaert frequency,

$$T_{M}=\frac{1}{f_{M}},$$

where $f_{M}$ was calculated using the volume-equivalent maximum radius. The resulting Minnaert duration was then compared with the experimentally measured collapse-associated timescale obtained from the interval between the two PVDF peaks.

The Minnaert duration and the bubble collapse duration showed a mean difference of 142.83 μs, with an $R^{2}$ value of 0.766. Although the two quantities are not expected to coincide exactly, because the Minnaert equation describes the small-amplitude oscillation of a spherical bubble, whereas the present laser-induced bubble undergoes strongly nonlinear growth and collapse near a solid boundary, their comparison provides an experimental assessment of whether the Minnaert frequency represents an appropriate characteristic timescale for the present analysis.

The dynamic mechanical properties of the polymers were obtained from published DMA data. The data sources were selected separately for polyethylene, PTFE, polyamide, and antistatic PET. Where the original sources presented the results graphically or as discrete measurements, power-law functions were fitted to the reported data to estimate the storage and loss moduli at $f_{M}$. Therefore, the equations presented below are fitted relations derived from the source data and are not equations originally proposed by the cited authors.

The viscoelastic response was expressed using the complex modulus,

(14) $$E^{*}\left(f\right)=E^{\prime }\left(f\right)+iE^{\prime \prime }\left(f\right),$$

where $E^{*}$ is the complex modulus, $E^{\prime }$ is the storage modulus, $E^{\prime \prime }$ is the loss modulus, and $i=\sqrt{-1}$. The storage modulus represents the elastic energy stored during cyclic deformation, whereas the loss modulus represents the energy dissipated through viscous and molecular motion [29].

#### 2.6.1. Storage Modulus

The frequency-dependent storage-modulus relations for polyethylene, PTFE, polyamide, and antistatic PET were obtained from published DMA data [27,30,31,32].

Power-law functions were fitted in the general form

(15) $$E^{\prime }\left(f\right)=E_{\mathrm{ref}}^{\prime }{\left(\frac{f}{f_{\mathrm{ref}}}\right)}^{n},$$

where $E_{\mathrm{ref}}^{\prime }$ is the storage modulus at the reference frequency, $f_{\mathrm{ref}}$ = 10,000 Hz, and *n* is the fitted frequency exponent. The resulting relations were

(16) $$\begin{array}{cc} E_{\mathrm{PE}}^{\prime }\left(f\right) & =1.10{\left(\frac{f}{10000}\right)}^{0.105}, \end{array}$$

(17) $$\begin{array}{cc} E_{\mathrm{PTFE}}^{\prime }\left(f\right) & =0.55{\left(\frac{f}{10000}\right)}^{0.135}, \end{array}$$

(18) $$\begin{array}{cc} E_{\mathrm{PA}}^{\prime }\left(f\right) & =2.80{\left(\frac{f}{10000}\right)}^{0.072}, \end{array}$$

(19) $$\begin{array}{cc} E_{\mathrm{APET}}^{\prime }\left(f\right) & =3.20{\left(\frac{f}{10000}\right)}^{0.097}. \end{array}$$

In these equations, *f* is expressed in Hz and *f*/10,000 is dimensionless. The storage modulus is expressed in GPa.

#### 2.6.2. Loss Modulus

The loss-modulus data were obtained from the same DMA sources used for the storage-modulus relations [27,30,31,32]. The fitted relations were expressed as

(20) $$E^{\prime \prime }\left(f\right)=E_{\mathrm{ref}}^{\prime \prime }{\left(\frac{f}{f_{\mathrm{ref}}}\right)}^{n},$$

where $E_{\mathrm{ref}}^{\prime \prime }$ is the loss modulus at $f_{\mathrm{ref}}$ = 10,000 Hz. The resulting equations were

(21) $$\begin{array}{cc} E_{\mathrm{PE}}^{\prime \prime }\left(f\right) & =0.044{\left(\frac{f}{10000}\right)}^{0.105}, \end{array}$$

(22) $$\begin{array}{cc} E_{\mathrm{PTFE}}^{\prime \prime }\left(f\right) & =0.044{\left(\frac{f}{10000}\right)}^{0.135}, \end{array}$$

(23) $$\begin{array}{cc} E_{\mathrm{PA}}^{\prime \prime }\left(f\right) & =0.168{\left(\frac{f}{10000}\right)}^{0.072}, \end{array}$$

(24) $$\begin{array}{cc} E_{\mathrm{APET}}^{\prime \prime }\left(f\right) & =0.128{\left(\frac{f}{10000}\right)}^{0.097}. \end{array}$$

The loss modulus is expressed in GPa. A higher value of $E^{\prime \prime }$ indicates greater energy dissipation under dynamic loading.

#### 2.6.3. Loss Factor

The loss factor was calculated from the ratio of the loss modulus to the storage modulus:

(25) $$\tan\delta \left(f\right)=\frac{E^{\prime \prime }\left(f\right)}{E^{\prime }\left(f\right)},$$

where δ is the phase angle between the cyclic stress and strain responses. The loss factor is dimensionless and represents the relative contribution of viscous damping to elastic energy storage [29].

Because the fitted storage- and loss-modulus relations for each material use the same frequency exponent, the frequency terms cancel. The resulting loss factors are

(26) $$\begin{array}{cc} \tan\delta_{\mathrm{PE}} & =\frac{0.044}{1.10}=0.040, \end{array}$$

(27) $$\begin{array}{cc} \tan\delta_{\mathrm{PTFE}} & =\frac{0.044}{0.55}=0.080, \end{array}$$

(28) $$\begin{array}{cc} \tan\delta_{\mathrm{PA}} & =\frac{0.168}{2.80}=0.060, \end{array}$$

(29) $$\begin{array}{cc} \tan\delta_{\mathrm{APET}} & =\frac{0.128}{3.20}=0.040. \end{array}$$

Within the fitted frequency range, PTFE had the highest calculated loss factor, followed by polyamide, while polyethylene and antistatic PET had the same calculated value.

#### 2.6.4. Relaxation Time

A relaxation time constant was estimated using the Kelvin–Voigt representation. The complex modulus is written as

(30) $$E^{*}=E^{\prime }+i\omega \eta ,$$

where η is the effective viscosity and ω is the angular frequency:

(31) ω=2πf.

Since

(32) $$E^{\prime \prime }=\omega \eta ,$$

the effective viscoelastic time constant was calculated as

(33) $$\tau =\frac{\eta }{E^{\prime }}=\frac{E^{\prime \prime }}{\omega E^{\prime }}=\frac{\tan\delta }{2\pi f}.$$

Using the bubble natural frequency gives

(34) $$\tau =\frac{\tan\delta }{2\pi f_{M}},$$

where τ is expressed in seconds. The calculated value was interpreted as a relaxation time constant.

### 2.7. Input Variables and Physical Parameters

The experimental analysis and impact-load prediction used 13 input variables representing bubble dynamics, test conditions, acoustic characteristics, and mechanical and viscoelastic properties of the polymer specimens, as summarized in Table 5.

The water properties were not used as model inputs because all experiments were conducted in the same surrounding liquid under the same room-temperature and atmospheric-pressure conditions. Therefore, water density, liquid viscosity, surface tension, and ambient pressure were treated as fixed experimental conditions rather than variables in the predictive model.

The bubble and experimental variables included the dimensional bubble-to-surface distance, *h*, maximum bubble radius, collapse time, and laser pulse energy. The normalized stand-off distance, γ, was not used directly. Instead, *h* was selected to preserve the actual length scale and avoid combining the distance and bubble-radius information before model training.

The material-related variables were speed of sound, combined acoustic impedance, flexural modulus, storage modulus, loss modulus, loss factor, effective viscoelastic time constant, yield strength, and solid density. These variables describe stress-wave transmission, stiffness, elastic energy storage, viscous dissipation, time-dependent response, deformation resistance, and material inertia.

Overall, the selected inputs represent the main bubble, experimental, acoustic, and material factors governing bubble-induced loading. The measured peak hydrodynamic impact load was used as the prediction target.

## 3. Machine Learning Models

Five regression methods were evaluated for predicting the peak impact load: linear regression, ridge regression, LASSO regression, random forest regression, and a multilayer perceptron (MLP). These methods represent unregularized linear, regularized linear, tree-based ensemble, and nonlinear neural-network approaches. The variables described in Section 2.7 were used as model inputs, while the measured peak impact load was used as the prediction target.

### 3.1. Linear Regression

Linear regression was used as the reference model for evaluating whether the peak impact load could be represented by a linear combination of the input variables. The prediction is expressed as

(35) $$\hat{y}=\beta_{0}+\sum_{j=1}^{p}\beta_{j}x_{j},$$

where $\hat{y}$ is the predicted impact load, $x_{j}$ is the *j*th input variable, $\beta_{0}$ is the intercept, and $\beta_{j}$ is the corresponding regression coefficient. The coefficients were estimated using ordinary least squares.

### 3.2. Ridge Regression

Ridge regression was applied to reduce coefficient instability caused by correlations among the input variables. The method adds an $L_{2}$ penalty to the least-squares objective:

(36) $$L_{\mathrm{ridge}}=\sum_{i=1}^{n}{\left(y_{i}-{\hat{y}}_{i}\right)}^{2}+\lambda \sum_{j=1}^{p}\beta_{j}^{2},$$

where λ controls the degree of coefficient shrinkage. Ridge regression reduces large coefficient variations without removing variables from the model.

### 3.3. LASSO Regression

LASSO regression was used to combine prediction with variable selection. It introduces an $L_{1}$ penalty into the least-squares objective:

(37) $$L_{\mathrm{LASSO}}=\sum_{i=1}^{n}{\left(y_{i}-{\hat{y}}_{i}\right)}^{2}+\lambda \sum_{j=1}^{p}\left|\beta_{j}\right|,$$

where λ controls the penalty strength. The $L_{1}$ penalty can reduce weak regression coefficients to zero and therefore identify a smaller set of input variables.

### 3.4. Random Forest Regression

Random forest regression was used to represent nonlinear relationships and interactions among the experimental and material variables. The method constructs multiple decision trees using bootstrap samples and random subsets of the input variables. The final prediction is obtained by averaging the individual tree predictions:

(38) $$\hat{y}=\frac{1}{B}\sum_{b=1}^{B}T_{b}\left(x\right),$$

where *B* is the number of trees and $T_{b}\left(x\right)$ is the prediction generated by the *b*th tree. The averaging process reduces prediction variance and limits overfitting.

### 3.5. Multilayer Perceptron

The multilayer perceptron was used to model nonlinear relationships between the input variables and the peak impact load. The network consisted of an input layer, one or more hidden layers, and a single regression output. For one hidden layer, the prediction can be written as

(39) $$\hat{y}=W_{2}\phi \left(W_{1}x+b_{1}\right)+b_{2},$$

where x is the input vector, $W_{1}$ and $W_{2}$ are the network weights, $b_{1}$ and $b_{2}$ are the bias vectors, and ϕ is the hidden-layer activation function. The network parameters were learned through backpropagation by minimizing the regression loss.

### 3.6. Bayesian Hyperparameter Optimization

Bayesian optimization was used to determine the hyperparameters of ridge regression, LASSO regression, random forest regression, and the MLP. Linear regression was excluded from the optimization because its coefficients were estimated directly using ordinary least squares.

The optimization objective was to identify the hyperparameter configuration that minimized the validation root mean square error:

(40) $$\lambda^{*}=\arg\min_{\lambda \in \Lambda }{\mathrm{RMSE}}_{\mathrm{val}}\left(\lambda \right),$$

where λ represents one hyperparameter configuration and Λ denotes the defined search space. Bayesian optimization used the results of previous trials to select the next configuration. This procedure reduced the number of unnecessary model evaluations compared with an exhaustive grid search. The Bayesian optimization settings listed in Table 6 were used for the machine learning analysis. The search space for the Bayesian optimization parameters, summarized in Table 7, was applied consistently throughout the analysis.

### 3.7. Five-Fold Training and Testing Procedure

To evaluate the prediction performance of the regression models, a five-fold cross-validation procedure was used. The full dataset consisted of 180 measurements obtained from 60 experimental conditions, corresponding to four polymer materials, three laser pulse energies, and five normalized stand-off distances. Each experimental condition was repeated three times.

To avoid data leakage, repeated measurements with the same input condition were not separated between the training and testing sets. In other words, data points having the same material, laser pulse energy, and stand-off distance were treated as one experimental group, even when the measured impact load showed small differences among repetitions. All repeated measurements belonging to the same group were assigned to the same fold. Therefore, a condition used for testing in one fold was not included in the training data for that fold.

In each cross-validation iteration, four folds were used for model training and the remaining fold was used for testing. This procedure was repeated five times so that each fold was used once as the testing set. The final model performance was calculated as the average value of the evaluation metrics over the five folds. This grouped cross-validation procedure provides a more reliable estimate of model generalization because the model is tested on experimental conditions that were not included during training.

### 3.8. Machine Learning Evaluation Metrics

The predictive performance of the machine learning models was evaluated using the root mean squared error (RMSE), mean absolute error (MAE), coefficient of determination ($R^{2}$), and mean absolute percentage error (MAPE). These metrics quantify the differences between the experimentally measured values and the values predicted by the model.

Let $y_{i}$ denote the actual value, ${\hat{y}}_{i}$ denote the predicted value, $\bar{y}$ denote the mean of the actual values, and *n* denote the total number of observations.

#### 3.8.1. Root Mean Squared Error

The root mean squared error measures the square root of the average squared difference between the actual and predicted values. It is expressed as

(41) $$\mathrm{RMSE}=\sqrt{\frac{1}{n}\sum_{i=1}^{n}{\left(y_{i}-{\hat{y}}_{i}\right)}^{2}}.$$

Because the prediction errors are squared, RMSE assigns a greater penalty to large errors than to small errors. Therefore, it is particularly useful when large prediction deviations are considered undesirable. RMSE has the same physical unit as the target variable, and a lower RMSE indicates better predictive performance.

#### 3.8.2. Mean Absolute Error

The mean absolute error calculates the average absolute difference between the actual and predicted values. It is defined as

(42) $$\mathrm{MAE}=\frac{1}{n}\sum_{i=1}^{n}\left|y_{i}-{\hat{y}}_{i}\right|.$$

Unlike RMSE, MAE treats all prediction errors proportionally and is less sensitive to large individual errors or outliers. MAE is expressed in the same physical unit as the target variable. A lower MAE represents a smaller average prediction error and, consequently, better model performance.

#### 3.8.3. Coefficient of Determination

The coefficient of determination evaluates the proportion of variance in the actual data that can be explained by the prediction model. It is calculated as

(43) $$R^{2}=1-\frac{\sum_{i=1}^{n}{\left(y_{i}-{\hat{y}}_{i}\right)}^{2}}{\sum_{i=1}^{n}{\left(y_{i}-\bar{y}\right)}^{2}},$$

where the mean of the actual values is given by

(44) $$\bar{y}=\frac{1}{n}\sum_{i=1}^{n}y_{i}.$$

An $R^{2}$ value close to 1 indicates that the model explains a large proportion of the variation in the target variable. An $R^{2}$ value of 0 indicates that the model performs similarly to a model that always predicts the mean value. A negative $R^{2}$ value indicates that the model performs worse than simply using the mean of the actual observations as the prediction.

#### 3.8.4. Mean Absolute Percentage Error

The mean absolute percentage error expresses the average absolute prediction error as a percentage of the actual value. It is defined as

(45) $$\mathrm{MAPE}=\frac{100}{n}\sum_{i=1}^{n}\left|\frac{y_{i}-{\hat{y}}_{i}}{y_{i}}\right|.$$

MAPE provides a scale-independent measure that facilitates comparison across datasets or target variables with different magnitudes. A lower MAPE indicates better predictive accuracy. However, MAPE is undefined when an actual value is zero and can become excessively large when the actual value is close to zero. Therefore, its application should be limited to datasets containing nonzero actual values or accompanied by an appropriate numerical treatment for values near zero.

Overall, lower RMSE, MAE, and MAPE values indicate better agreement between the predicted and actual values, whereas a higher $R^{2}$ value indicates stronger explanatory and predictive capability.

## 4. Results and Discussion

### 4.1. Comparative Performance of Regression Models

Five regression models were tested using all 13 input variables to predict the peak impact load caused by bubble collapse. The models were random forest regression, multilayer perceptron (MLP) regression, lasso regression, linear regression, and ridge regression.

Before model comparison, the main hyperparameters of the random forest, MLP, lasso, and ridge regression models were tuned using Bayesian optimization. Linear regression was included as a baseline model and evaluated using ordinary least squares without hyperparameter optimization.

The model performance was evaluated using the root mean squared error (RMSE), mean absolute error (MAE), coefficient of determination ($R^{2}$), and mean absolute percentage error (MAPE). Lower RMSE, MAE, and MAPE values mean smaller prediction errors, while an $R^{2}$ value closer to one means that the model explains more of the variation in the measured impact load. Bayesian optimization was used to tune the hyperparameters of each machine learning model before comparing their regression performance. The optimization configuration, including the sampler, objective function, cross-validation strategy, and number of trials, is summarized in Table 6. The Bayesian hyperparameter search space is presented in Table 7, and the resulting optimized hyperparameters are reported in Table 8. The regression analysis was conducted using grouped five-fold cross-validation to ensure that samples from the same group were not shared between the training and validation sets.

The machine learning regression results are summarized in Table 9. Among the five models, random forest regression gave the best overall performance. It achieved the lowest RMSE and MAE values, 0.6820 and 0.5339, respectively, and the highest $R^{2}$ value of 0.9168. This result indicates that the optimized random forest model explained about 91.68% of the variation in the measured impact load and produced the smallest prediction errors among the tested models.

The MLP regressor gave the second-best result, with an RMSE of 0.7183, an MAE of 0.5651, an $R^{2}$ value of 0.9127, and a MAPE of 13.66%. Although the MLP model also showed strong predictive performance, the random forest model performed slightly better in terms of RMSE, MAE, $R^{2}$, and MAPE. Therefore, random forest regression was selected as the best-performing model overall.

The optimized Random Forest model achieved a MAPE of 12.44%, indicating a mean absolute percentage deviation of approximately 12% between the predicted and measured impact loads. However, no generally established accuracy threshold is available for the present single-bubble impact-load prediction problem. Therefore, this value should not be interpreted as a universal engineering-acceptance criterion. Rather, together with the RMSE, MAE, and $R^{2}$ results, the present MAPE demonstrates the feasibility of predicting impact-load trends within the investigated experimental parameter space.

The good performance of the random forest and MLP models suggests that the relationship between the input variables and the impact load is nonlinear. The impact load generated by bubble collapse depends on several coupled factors, including bubble size, collapse time, stand-off distance, laser energy, material properties, viscoelastic properties, and acoustic properties. These variables may interact with each other and may not contribute to the impact load in a purely linear manner. Random forest regression can capture such nonlinear effects through an ensemble of decision trees, while the MLP model can represent nonlinear relationships through its hidden layers.

The linear models showed lower predictive performance than the nonlinear models. Ridge regression gave an RMSE of 1.0713, an MAE of 0.8461, an $R^{2}$ value of 0.7994, and a MAPE of 20.44%. Lasso regression gave an RMSE of 1.0715, an MAE of 0.8455, an $R^{2}$ value of 0.7995, and a MAPE of 20.37%. Linear regression gave an RMSE of 1.0725, an MAE of 0.8444, an $R^{2}$ value of 0.7989, and a MAPE of 20.31%. These results indicate that a simple linear combination of the input variables was less effective in describing the impact-load behavior.

This difference in performance may be attributed to nonlinear interactions among the physical variables. In particular, the effects of storage modulus, loss modulus, stand-off distance, bubble radius, collapse time, laser energy, and acoustic impedance may vary depending on the experimental condition. This may explain why the nonlinear models, especially random forest regression and the MLP regressor, provided better predictions.

**Figure 6:**
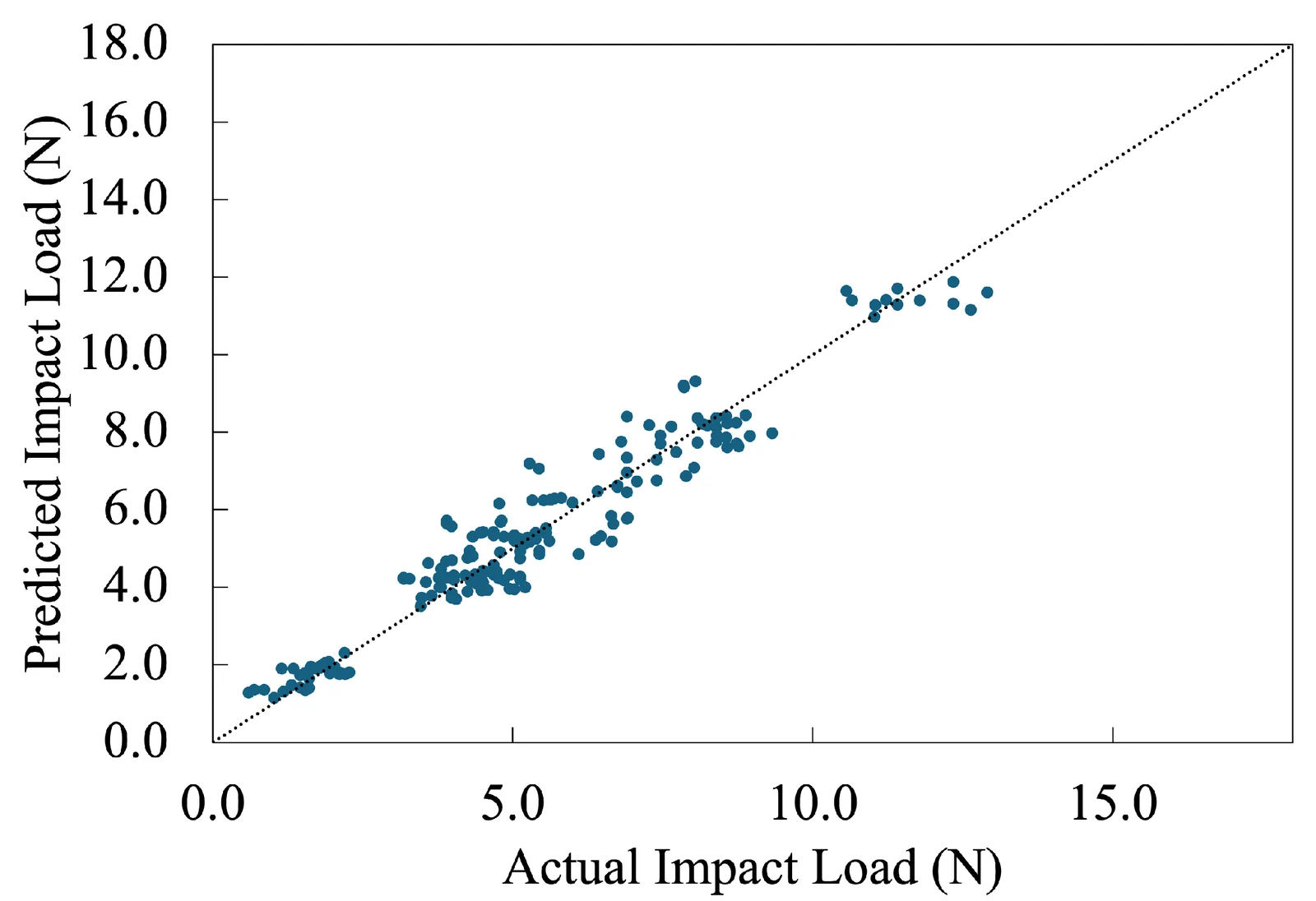
Comparison between experimentally measured and predicted bubble-collapse impact loads using the random forest regression model. The data points close to the one-to-one reference line indicate good agreement between the measured and predicted values.

The prediction performance of the best-performing model, random forest regression, is further illustrated in Figure 6. The scatter plot compares the experimentally measured impact loads with the values predicted by the random forest model. Most data points are distributed close to the one-to-one reference line, indicating good agreement between the measured and predicted values. This result is consistent with the quantitative performance metrics reported in Table 9, where random forest regression achieved the lowest RMSE and MAE and the highest $R^{2}$ value among the evaluated models. Therefore, the random forest model was selected for the subsequent feature-importance and SHAP-based analyses.

**Figure 7:**
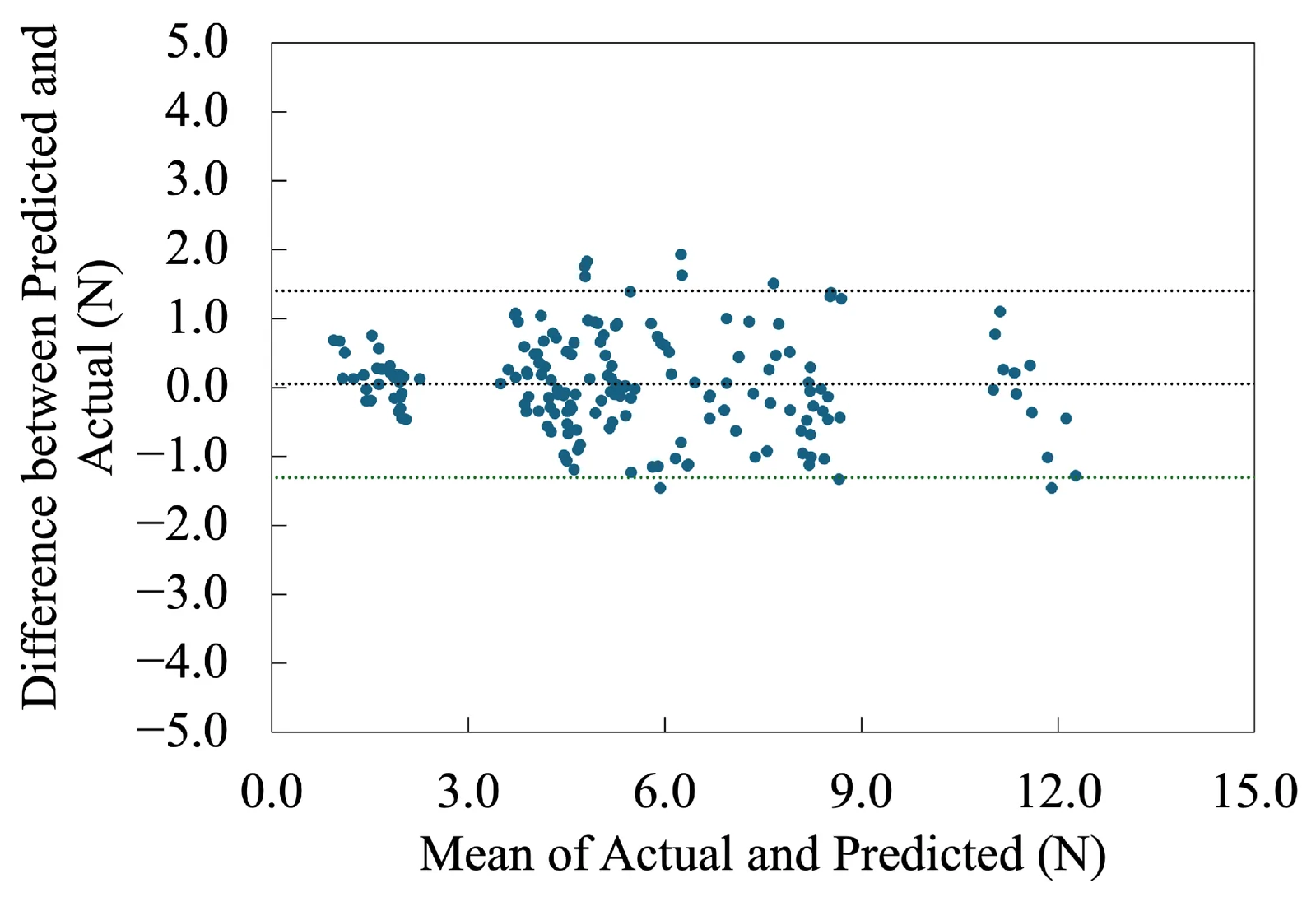
Bland–Altman plot comparing the predicted and actual values.

The Bland–Altman plot shown in Figure 7 indicates good agreement between the predicted and actual values, with a small mean difference of 0.050. The 95% limits of agreement ranged from −1.303 to 1.403. Although the overall bias was minimal, the wider scatter at higher mean values suggests increased prediction variability for larger measurements.

The effect of the cross-validation method was examined to assess the stability of the regression results. Using the Random Forest model in Table 9 and the Bayesian optimized parameters in Table 8, grouped five fold cross-validation, repeated grouped five fold cross-validation, and leave one condition out cross-validation were compared, as summarized in Table 10. For repeated grouped five fold cross-validation, the grouped split was repeated 20 times, giving 100 validation folds in total. In all cases, the three repeated trials belonging to the same experimental condition were kept in the same fold.

Grouped five fold cross-validation was used as the main validation method because it gave lower errors than repeated grouped five fold cross-validation. The grouped five fold result gave an RMSE of 0.6820, MAE of 0.5339, $R^{2}$ of 0.9168, and MAPE of 12.44%. The repeated grouped five fold result gave an RMSE of 0.7518, MAE of 0.5624, $R^{2}$ of 0.9200, and MAPE of 13.59%. These results show that the selected grouped five fold split gave strong prediction accuracy while still avoiding separation of repeated trials from the same condition.

Leave one condition out cross-validation gave an RMSE of 0.6720, MAE of 0.5079, $R^{2}$ of 0.9361, and MAPE of 11.88%. Although this result was slightly better, it was treated as a robustness check rather than the main validation method. This is because each test fold contains only one condition, or three repeated measurements, so the fold level results can be sensitive to the variability within a single condition. In addition, if this method is used for both hyperparameter tuning and final reporting without a nested procedure, the final estimate may become biased. Therefore, grouped five fold cross-validation was retained as the main validation method, while repeated grouped five fold and leave one condition out cross-validation were used to check the stability of the selected model.

The model was developed to estimate cavitation induced impact load under different experimental conditions, not to replace cavitation erosion lifetime tests. The MAPE of 12.44% from grouped five fold cross-validation indicates that the model can provide a useful estimate of impact load for preliminary load estimation, trend analysis, and material screening.

However, this error should not be interpreted as the error of fatigue-life prediction itself. Fatigue life is highly sensitive to the applied load, especially near the fatigue limit, and a load prediction error may cause a much larger difference in the estimated lifetime. In addition, the present model predicts only the load and does not directly model fatigue crack initiation, local plastic deformation, crack growth, or cavitation-erosion damage evolution. Therefore, the model is useful for comparing the relative severity of experimental conditions, but fatigue-life evaluation or final engineering design should still include safety factors and confirmatory fatigue or cavitation-erosion tests.

**Figure 8:**
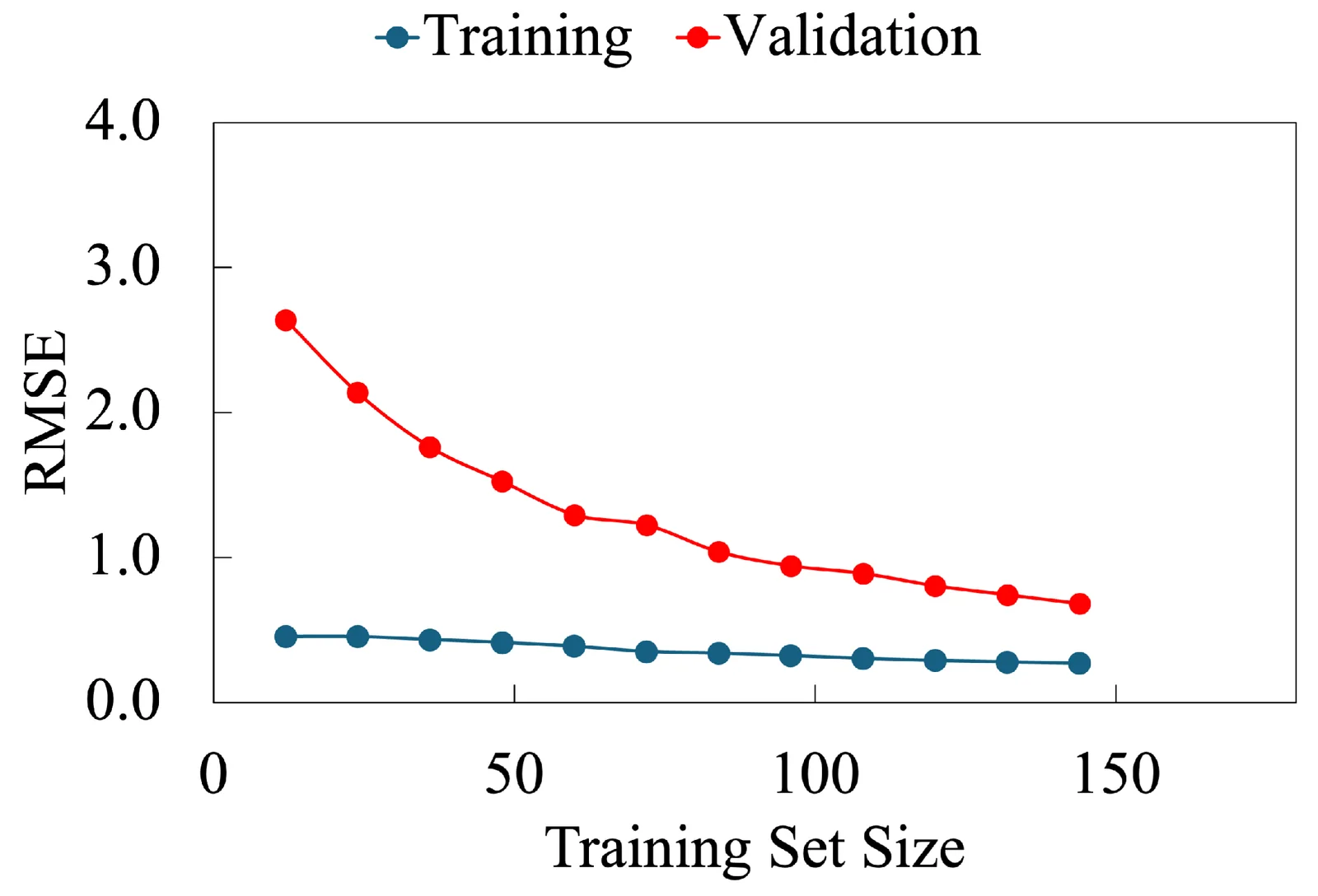
Learning curve of the Random Forest regression model showing the training and validation RMSE as a function of training set size.

The learning curve in Figure 8 shows a persistent gap between the training and validation RMSE values, indicating residual model variance and some degree of overfitting. However, the validation RMSE continues to decrease as the training-set size increases, while the training and validation curves gradually approach each other. This behavior suggests that the present dataset is not yet sufficient to establish fully converged generalization performance and that additional experimental data are likely to improve model robustness and reduce prediction variance. Accordingly, the prediction accuracy reported in this study should be interpreted within the investigated experimental parameter space rather than as evidence of universal generalization to arbitrary materials or cavitation conditions.

For practical hydraulic applications, the proposed single-bubble collapse model cannot be directly extended to multiple-bubble or cloud cavitation without considering additional phenomena such as bubble population density, collapse frequency, bubble–bubble interaction, cloud shedding, pressure-wave interaction, and repeated impact accumulation. Cloud cavitation can produce stronger and more spatially distributed loading because interactions among neighboring bubbles and cavity structures may enhance collective collapse and shock-wave focusing [33]. Hydrodynamic cavitation studies in Venturi-type reactors have also shown that cavitation behavior depends strongly on flow conditions, cavitation number, Reynolds number, and thermodynamic effects [34].

The present study therefore addresses a more fundamental scale of the problem: the local impulsive loading generated by an individual bubble-collapse event near a polymer surface. Such single-bubble experiments provide controlled conditions for examining how bubble dynamics and target-material properties affect the measured transient load. However, the subsequent accumulation of plastic deformation, crack initiation and growth, and material removal under repeated cavitation impacts are outside the direct scope of the present model.

Extension of the present framework to multi-bubble or cloud-cavitation conditions requires additional descriptors that are not included in the current single-bubble model. In the present experiments, each collapse event can be characterized by well-defined quantities such as maximum bubble radius, collapse time, and stand-off distance. In multi-bubble cavitation, however, bubble size, position, collapse timing, and deformation become spatially and temporally distributed, and neighboring bubbles can interact through pressure waves and surrounding liquid motion. Collective collapse may therefore produce loading that cannot be represented by a simple superposition of independent single-bubble impacts.

A future multi-bubble extension of the present data-driven framework would require statistical and population-based descriptors, such as bubble-size distribution, bubble number density, spatial distribution, collapse-frequency distribution, and cloud-scale void fraction. Shape-related descriptors may also be required to account for asymmetric and non-spherical collapse. Such descriptors could be combined with the material-response parameters identified in the present study to examine how stochastic bubble-cloud dynamics are translated into transient and cumulative loading of solid surfaces.

The interaction of multiple bubbles in other applications, such as airgun-bubble arrays used to modify low-frequency pressure-wave characteristics, similarly illustrates that bubble–bubble interaction can substantially alter the resulting pressure field [33]. However, the present study is limited to controlled single-bubble collapse, and validation of a multi-bubble extension will require experiments in which bubble populations and collective collapse behavior are directly characterized.

### 4.2. Correlation of the Input Variables

**Figure 9:**
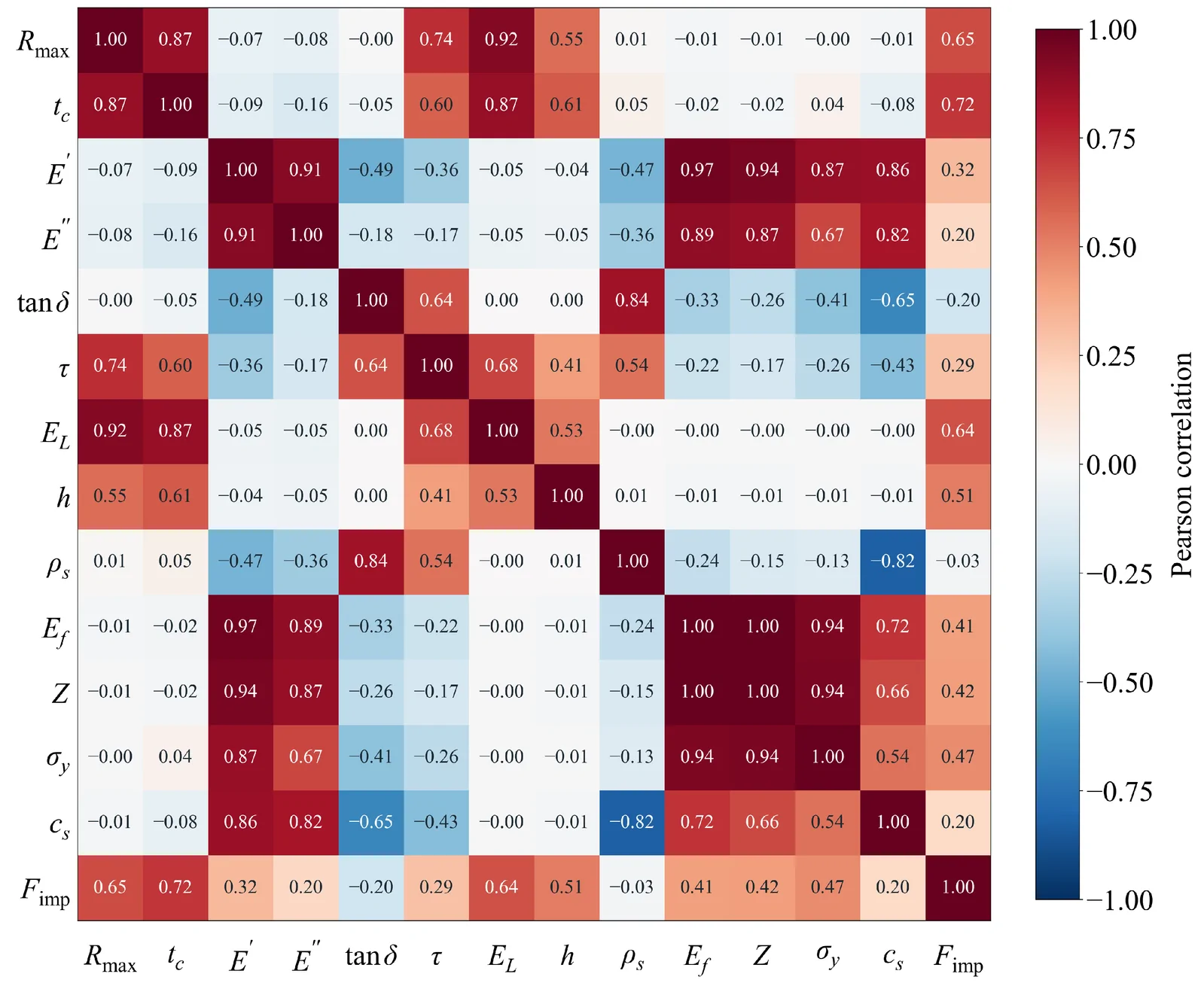
Pearson correlation matrix of the input variables and output response quantities.

Figure 9 shows the Pearson correlation matrix of the selected input variables and the impact load. The Pearson coefficient between two variables $x_{i}$ and $x_{j}$ is defined as

(46) $$r_{ij}=\frac{\mathrm{cov}(x_{i},x_{j})}{\sigma_{x_{i}}\sigma_{x_{j}}},$$

where $r_{ij}=1$ indicates a perfect positive linear correlation, $r_{ij}=-1$ indicates a perfect negative linear correlation, and $r_{ij}\approx 0$ indicates little or no linear correlation.

The dominant correlations are observed among the maximum bubble radius $R_{\max}$, collapse time $t_{c}$, laser pulse energy $E_{L}$, and relaxation time τ. The maximum bubble radius and collapse time are strongly correlated,

$$r(R_{\max},t_{c})=0.87,$$

which is physically reasonable because larger bubbles generally require a longer time to collapse. The laser pulse energy is also strongly correlated with both bubble-response quantities:

$$r\left(E_{L},R_{\max}\right)=0.92,\;r\left(E_{L},t_{c}\right)=0.87.$$

This indicates that increasing laser energy promotes the formation of larger cavitation bubbles and longer collapse durations.

Physically, the laser pulse deposits energy into the liquid, generating a localized plasma and a rapidly expanding vapor cavity. If $E_{L}$ is the laser pulse energy and η is the fraction of energy converted into bubble energy, the absorbed energy can be written as

(47) $$E_{\mathrm{abs}}=\eta E_{L}.$$

A first-order energy balance for a spherical cavitation bubble gives

(48) $$E_{\mathrm{abs}}\approx \frac{4\pi }{3}R_{\max}^{3}\left(p_{\infty }-p_{v}\right)+4\pi R_{\max}^{2}\gamma ,$$

where $p_{\infty }$ is the ambient pressure, $p_{v}$ is the vapor pressure, and γ is the surface tension. When the pressure-volume work dominates the surface-energy term, the maximum bubble radius scales approximately as

(49) $$R_{\max}∼{\left(\frac{3\eta E_{L}}{4\pi (p_{\infty }-p_{v})}\right)}^{1/3}.$$

The collapse time follows the Rayleigh collapse relation,

(50) $$t_{c}=0.915R_{\max}\sqrt{\frac{\rho_{l}}{p_{\infty }-p_{v}}},$$

where $\rho_{l}$ is the liquid density. Therefore, the strong positive correlations among $E_{L}$, $R_{\max}$, and $t_{c}$ are consistent with cavitation-bubble physics.

The stand-off distance *h* also shows moderate positive correlations with $R_{\max}$, $t_{c}$, and $E_{L}$, with correlation coefficients of approximately 0.55, 0.61, and 0.53, respectively. This suggests that the bubble response is affected not only by laser energy but also by the geometric configuration of the experiment. The relaxation time τ is positively correlated with $R_{\max}$, $t_{c}$, and $E_{L}$, with coefficients of 0.74, 0.60, and 0.68, respectively, indicating that it is associated with the temporal evolution of the bubble.

The material-related variables show strong internal correlations. For example, the flexural modulus $E_{f}$, acoustic impedance *Z*, tensile yield strength $\sigma_{y}$, storage modulus $E^{\prime }$, and sound speed $c_{s}$ are highly correlated with each other. In particular,

$$r\left(E_{f},Z\right)=1.00,\;r\left(E_{f},\sigma_{y}\right)=0.94,\;r\left(E^{\prime },E_{f}\right)=0.97,\;r\left(E^{\prime },Z\right)=0.94.$$

These correlations indicate that several mechanical properties vary together in the present dataset. Therefore, their individual effects should be interpreted carefully, because Pearson correlation cannot separate the independent contribution of highly correlated predictors.

The impact load $F_{\mathrm{imp}}$ shows positive correlations with the main bubble-response variables:

$$r\left(F_{\mathrm{imp}},R_{\max}\right)=0.65,\;r\left(F_{\mathrm{imp}},t_{c}\right)=0.72,\;r\left(F_{\mathrm{imp}},E_{L}\right)=0.64,\;r\left(F_{\mathrm{imp}},h\right)=0.51.$$

These results indicate that larger bubbles, longer collapse times, higher laser energy, and larger stand-off distance are generally associated with higher measured impact load. This is physically reasonable because a larger bubble collapse can generate stronger pressure-wave emission and more energetic liquid motion near the solid surface.

In addition to the bubble-response variables, $F_{\mathrm{imp}}$ is also moderately correlated with several material-related variables, including

$$r\left(F_{\mathrm{imp}},\sigma_{y}\right)=0.47,\;r\left(F_{\mathrm{imp}},Z\right)=0.42,\;r\left(F_{\mathrm{imp}},E_{f}\right)=0.41,\;r\left(F_{\mathrm{imp}},E^{\prime }\right)=0.32.$$

This suggests that the measured impact load is influenced by both the cavitation dynamics and the mechanical/acoustic response of the polymer target. However, because many material properties are mutually correlated, Pearson correlation alone cannot determine causality or isolate the independent effect of each variable. Therefore, SHAP analysis was further conducted to quantify the nonlinear feature contributions in the trained random forest model.

### 4.3. Global Feature Importance and Stand-Off Distance Dependence

Shapley additive explanations (SHAP) was used to estimate how much each input variable affected the predictions of the random forest regression model. The variables were ranked according to their mean absolute SHAP values. A larger mean absolute SHAP value indicates that the variable had a stronger overall contribution to the predicted impact load.

Table 11 shows the updated feature ranking obtained from the SHAP analysis. The bubble collapse time, acoustic impedance, and loss modulus were the three most influential variables, with mean absolute SHAP values of 1.406, 1.050, and 0.549, respectively. These results show that bubble collapse time, acoustic impedance, and loss modulus were the three most influential predictors in the Random Forest model. Their combined importance suggests that impact-load prediction depends on contributions from bubble dynamics, acoustic coupling, and the dynamic material response, rather than on a single material property.

The SHAP ranking was then used for backward feature elimination. The model was first trained using all 13 input variables. After that, the variable with the lowest mean absolute SHAP value was removed, and the random forest model was retrained. The SHAP values were recomputed after each removal step, so the feature ranking was updated recursively during the elimination process. For a model with *n* retained features, the feature subset was written as

(51) $$S_{n}=\left\{f_{1},f_{2},\ldots ,f_{n}\right\},$$

where $f_{1}$ to $f_{n}$ denote the features retained at that elimination step.

**Figure 10:**
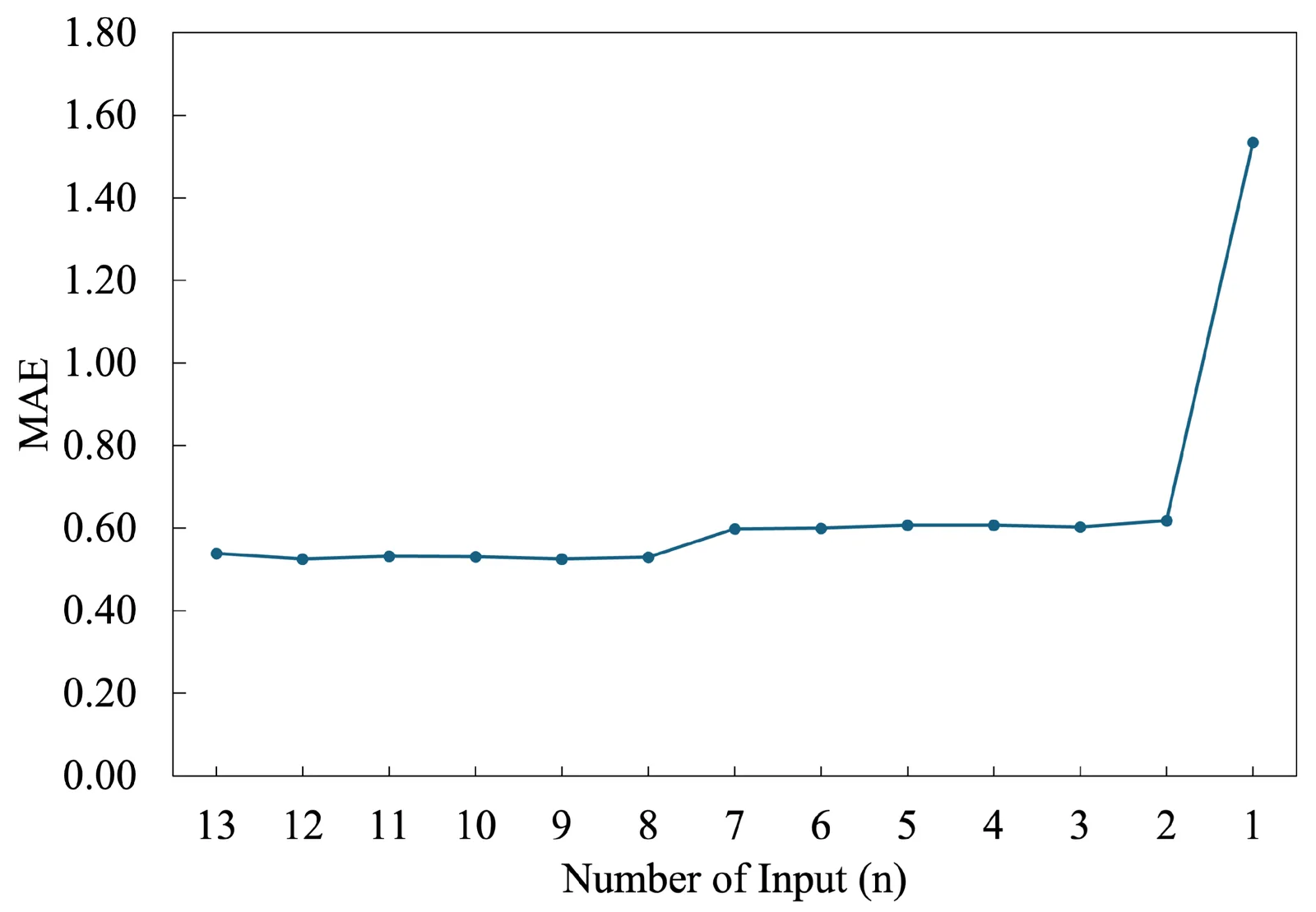
Change in mean absolute error during SHAP-guided backward feature elimination. The random forest model was retrained after each feature-removal step, and SHAP values were recomputed recursively. The MAE remained nearly unchanged down to approximately eight input variables, but increased markedly when the model was reduced to only one input.

Figure 10 shows the change in mean absolute error (MAE) as the number of input variables was reduced. The MAE remained nearly constant when the number of input variables was reduced from 13 to 8. This indicates that several low-ranked variables, including the loss factor, effective relaxation time, laser energy, flexural modulus, and sound speed, contributed only limited additional predictive information to the model.

A clear increase in MAE occurred when the number of variables was reduced from 8 to 7. This increase appeared after the distance-to-solid variable was removed, indicating that the stand-off geometry still provided useful information for predicting the impact load. From 7 to 2 input variables, the MAE remained relatively close, although slightly higher than that of the full model. However, when the model was reduced to only one input variable, the MAE increased sharply. This shows that the bubble collapse time alone was insufficient to describe the impact load, even though it was the most important individual feature.

The SHAP-guided feature-elimination results showed that the model could be reduced substantially without a major loss of predictive accuracy. The eight-variable subset retained most of the predictive capability of the full model and consisted of the bubble radius $R_{\max}$, bubble collapse time $t_{c}$, storage modulus $E^{\prime }$, loss modulus $E^{\prime \prime }$, distance to the solid boundary *h*, solid density $\rho_{s}$, acoustic impedance *Z*, and tensile yield strength $\sigma_{y}$. This result is physically reasonable because the impact load generated by bubble collapse depends on both the bubble dynamics and the mechanical response of the target material.

**Figure 11:**
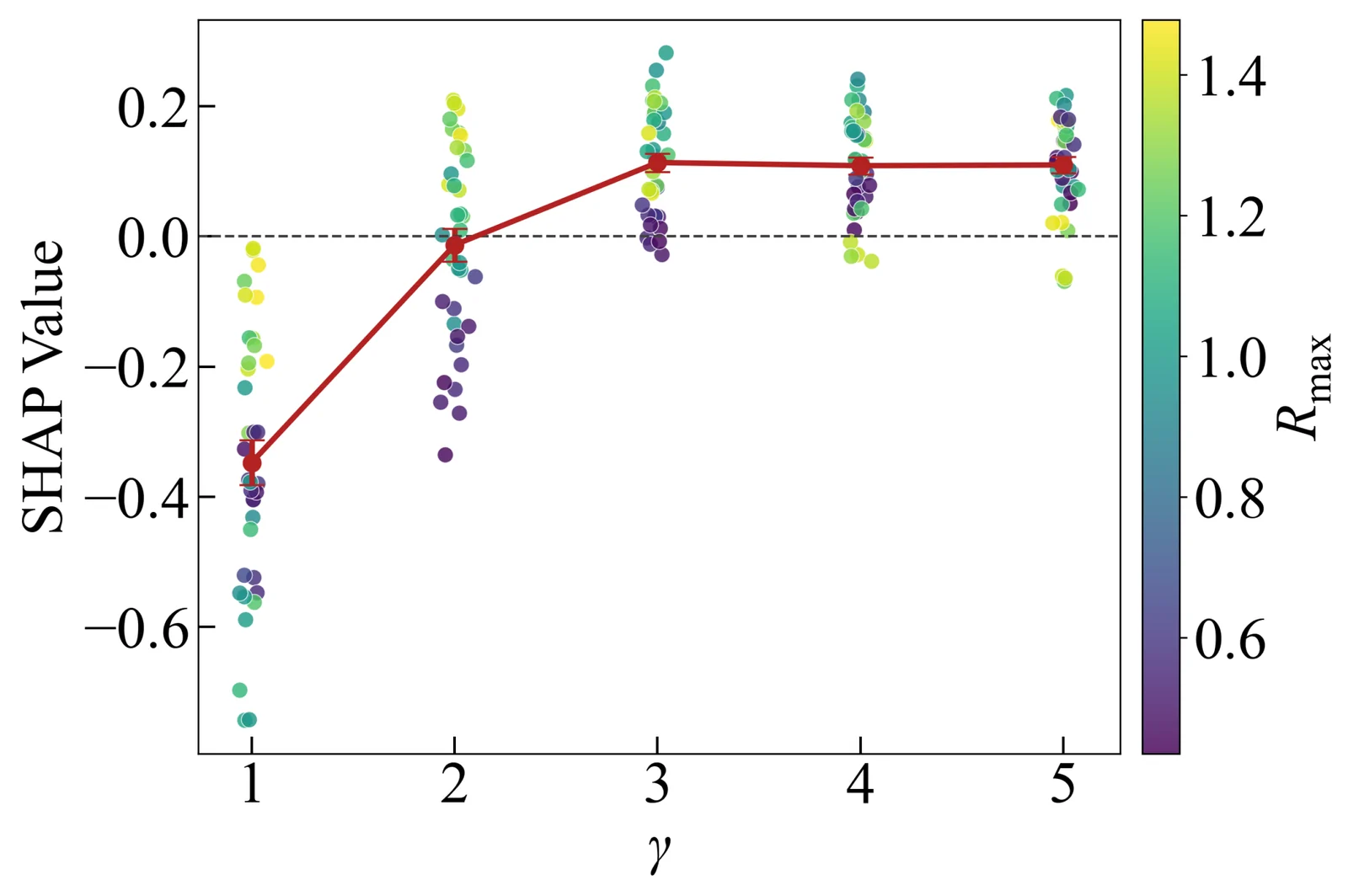
SHAP dependence plot of the stand-off distance. The horizontal axis represents the normalized stand-off distance $\gamma =h/R_{\max}$, and the vertical axis represents the SHAP value of the stand-off distance. The color scale indicates the maximum bubble radius $R_{\max}$. The red curve represents the mean SHAP value at each γ, with error bars showing the standard error of the mean.

To examine how the contribution of the dimensional distance feature *h* varies with the normalized stand-off condition, the SHAP value assigned to *h* was plotted against $\gamma =h/R_{\max}$, as shown in Figure 11. The horizontal axis represents the normalized stand-off distance, $\gamma =h/R_{\max}$, while the vertical axis represents the SHAP value of the stand-off distance. The color of each point indicates the maximum bubble radius $R_{\max}$. The red line shows the mean SHAP value at each value of γ, with the error bars representing the standard error of the mean.

The SHAP dependence plot indicates a nonlinear change in the contribution of stand-off distance, with distinct behavior at γ=1, a transition around γ=2, and a relatively plateau-like response for γ=3–5. This trend is qualitatively consistent with previously reported changes in bubble-collapse morphology and in the relative contributions of microjet and pressure-wave loading with stand-off distance. However, because microjet velocity and collapse-induced pressure-wave loading were not measured separately in the present experiments, the observed SHAP transition cannot be uniquely assigned to either mechanism.

The importance of $t_{c}$ and $R_{\max}$ in SHAP ranking reflects the role of cavitation dynamics, where larger and longer-lived bubbles can generate stronger collapse-induced pressure waves and liquid motion. Acoustic impedance, *Z*, is also physically meaningful because it governs the reflection and transmission of collapse-induced pressure waves at the liquid–solid interface. Firly et al. [35] reported that, for metals, bubble-collapse impact loads are strongly associated with acoustic impedance and elastic wave propagation, whereas for polymers the resulting deformation is strongly affected by yielding behavior. Similarly, Hattori and Itoh [36] showed that impact loads acting on plastic surfaces are lower than those on metals because plastics have relatively low acoustic impedance, *Z*. The importance of *h* is also consistent with single-bubble collapse near a wall, where the stand-off distance affects the relative contributions of microjet impact and shock-wave loading [10]. Meanwhile, the importance of $E^{\prime }$, $E^{\prime \prime }$, *Z*, $\rho_{s}$, and $\sigma_{y}$ suggests that stress-wave transmission, elastic response, viscous dissipation, and material resistance also influence the measured impact load. However, because several material properties are correlated with one another, the SHAP results should be interpreted as model-based feature contributions rather than direct causal effects.

### 4.4. Near-Wall Viscoelastic Response at γ=1

To further investigate the role of polymer viscoelasticity under near-wall collapse conditions, an additional analysis was performed using only the measurements obtained at γ=1. Because this subset contained only 36 measurements, the input space was reduced from 13 variables to five physically representative variables: maximum bubble radius $R_{\max}$, collapse time $t_{c}$, acoustic impedance *Z*, storage modulus $E^{\prime }$, and loss modulus $E^{\prime \prime }$. These variables represent bubble dynamics, acoustic coupling, elastic energy storage, and viscous dissipation.

The reduced five-feature Random Forest model yielded an RMSE of 1.2097N, MAE of 0.9339N, $R^{2}=0.7604$, and MAPE of 24.24%. Because this subset was substantially smaller than the full dataset, the reduced model was used primarily for mechanism-oriented interpretation rather than as an alternative predictive model to the main 180-measurement analysis.

To examine whether further reduction of the input space was possible, the five physically selected features were sequentially removed according to their feature importance, and the corresponding MAE was evaluated. The five-feature representation showed the lowest MAE among the tested reduced models, while the prediction error generally increased as additional variables were removed. Therefore, all five variables were retained for the subsequent near-wall feature-importance analysis.

Pearson correlation analysis showed that $F_{\mathrm{imp}}$ was moderately correlated with $R_{\max}$ (r=0.65) and $t_{c}$ (r=0.65), whereas weaker linear correlations were observed for *Z* (r=0.42), $E^{\prime }$ (r=0.32), and $E^{\prime \prime }$ (r=0.20). In contrast, the SHAP analysis of the reduced Random Forest model ranked $E^{\prime }$ and $E^{\prime \prime }$ as the two most influential predictors, followed by $R_{\max}$, $t_{c}$, and *Z*.

The difference between the Pearson correlation and SHAP results indicates that the contribution of the viscoelastic properties under the near-wall condition cannot be represented by a simple linear dependence. Storage modulus $E^{\prime }$ characterizes the recoverable elastic component of the polymer response and is associated with transient stiffness and stress transmission, whereas loss modulus $E^{\prime \prime }$ characterizes viscous energy dissipation and attenuation. The high SHAP importance of both quantities therefore suggests that the measured impact response under near-wall collapse conditions is sensitive to the balance between elastic stress transmission and viscoelastic dissipation.

Because strongly asymmetric collapse and enhanced wall-directed microjet interaction are expected at small stand-off distances, this balance may become more relevant near the solid boundary than under more distant collapse conditions. However, microjet and pressure-wave contributions were not separately measured in the present experiments, and the present analysis therefore does not establish a direct causal relationship between microjet loading and the increased importance of the viscoelastic properties.

Furthermore, $E^{\prime }$, $E^{\prime \prime }$, and *Z* are strongly correlated within the four-polymer dataset, and the γ=1 subset contains only 36 measurements. Therefore, the present analysis should be interpreted as exploratory evidence that viscoelastic properties collectively provide important predictive information under near-wall conditions, rather than as a quantitative separation of their individual causal effects.

### 4.5. Impact-Load Normalization Using Effective Acoustic Impedance

The measured impact load was normalized by the equivalent acoustic impedance, *Z*, defined in Equation (6). The normalized impact load was calculated as

$$F_{\mathrm{norm}}=\frac{F_{\mathrm{impact}}}{Z},$$

where $F_{\mathrm{impact}}$ is the measured impact load. This normalization enables comparison of the impact response among different polymer materials by accounting for differences in their equivalent acoustic impedance.

**Figure 12:**
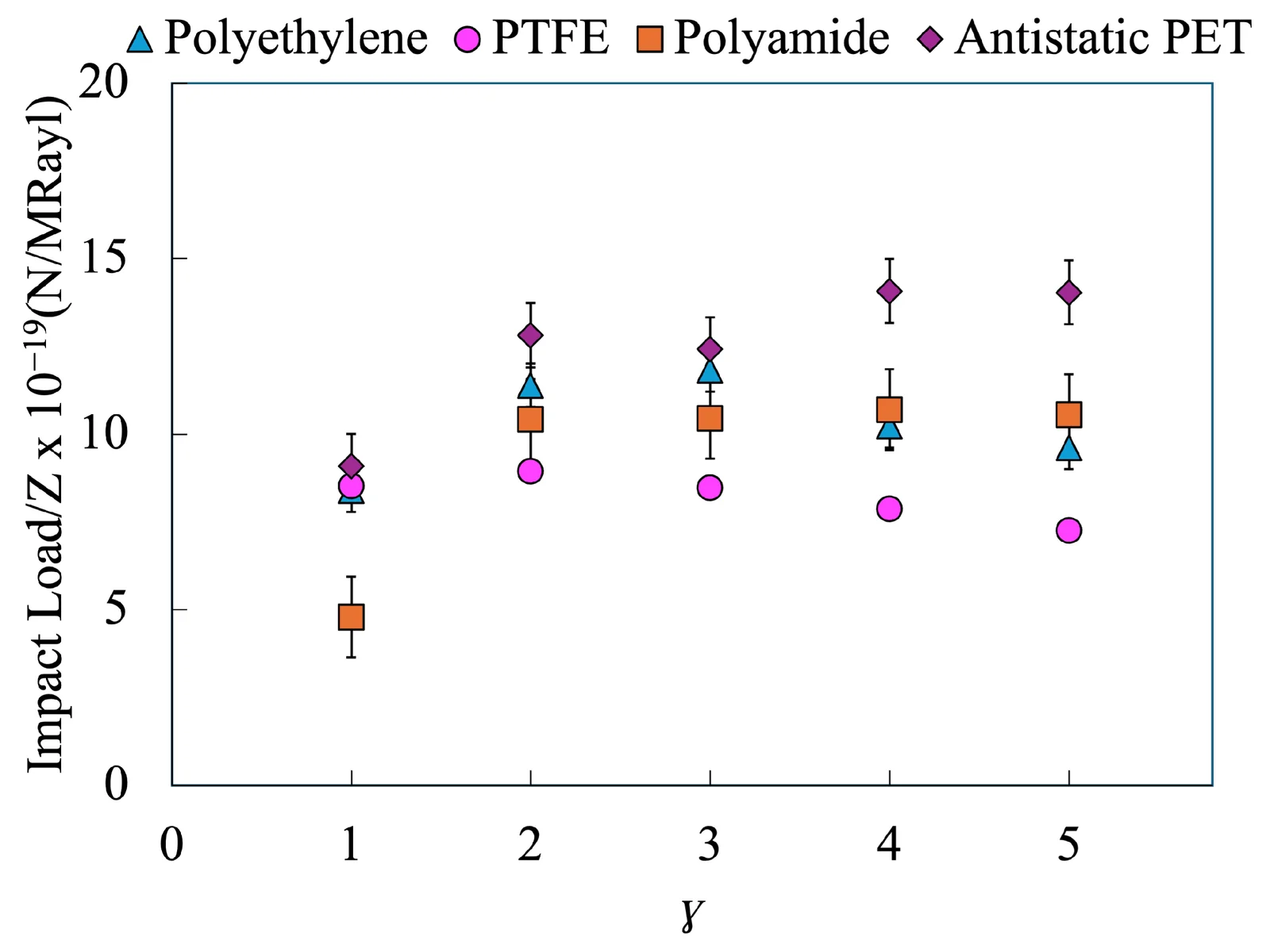
Impact load normalized by the equivalent acoustic impedance, *Z*, for the investigated polymer materials. The comparison indicates that the equivalent acoustic impedance accounts for part of the material-dependent variation in the measured impact load, while residual differences remain among the polymers. Error bars represent the variability of the repeated measurements.

As shown in Figure 12, the impact load normalized by *Z* exhibits material-dependent trends across the investigated conditions. Acoustic impedance accounts for part of the material-dependent variation but is insufficient as a universal scaling parameter. The combined SHAP analyses indicate that the measured impact load is better interpreted as a multivariable response involving bubble dynamics, acoustic coupling, viscoelastic response, and stand-off condition.

### 4.6. Study Limitations

A further limitation is the size and structure of the present dataset. Although 180 measurements were available, they represent 60 experimental conditions with three repeated measurements per condition. The additional cross-validation and learning-curve analyses indicate that the Random Forest model retains some variance associated with the limited training data. Moreover, the four investigated polymers cover only a limited range of polymer mechanical and viscoelastic properties. Therefore, the reported model performance should primarily be interpreted as prediction within the present experimental domain rather than as evidence of broad generalization. Additional materials and independent experimental conditions will be required to establish a more broadly applicable model and to determine whether the present prediction accuracy is sufficient for specific engineering applications.

To evaluate the generalization capability of the selected model for unseen polymer materials, leave-one-material-out cross-validation was performed. In this analysis, the Bayesian-optimized Random Forest Regression model was trained using data from three materials and tested on the remaining held-out material. The hyperparameters were kept fixed during this validation, and no additional feature selection or model tuning was performed.

The results showed that material-level generalization was more limited than the grouped 5-fold cross-validation performance. Across the four held-out materials, the RMSE ranged from 1.4641 to 2.6475, the MAE ranged from 1.2717 to 2.3515, and the MAPE ranged from 32.54% to 90.40%. The $R^{2}$ values ranged from −9.1593 to 0.6023. The negative $R^{2}$ obtained when PTFE was held out indicates that the model performed worse than predicting the mean response for that material, suggesting weak extrapolation capability for some unseen polymers. Therefore, although the selected Random Forest model performed well under grouped cross-validation, its prediction reliability decreases when the entire polymer material is excluded from training.

These results suggest that the present model is primarily valid within the material domain included in the training dataset and has limited extrapolation capability to unseen polymer materials. Therefore, further improvement of the model generalization performance will require additional training data, including a wider range of polymer materials and potentially different liquid environments. This limitation should be considered when applying the current model outside the experimental conditions used in this study. The substantial deterioration in prediction performance for the held-out polymers indicates that the present model has limited ability to extrapolate to materials whose mechanical and viscoelastic properties are not sufficiently represented in the training dataset. In particular, the negative $R^{2}$ values obtained for some held-out materials indicate that the high prediction accuracy obtained within the original four-material dataset should not be interpreted as evidence of general applicability to arbitrary polymer materials.

Although LOMO validation does not constitute validation using a completely independent external experimental dataset, it provides a stricter assessment of material-wise extrapolation than the grouped cross-validation used for the main model. The present results therefore define an important applicability boundary of the model and indicate that additional polymer materials spanning a broader range of acoustic, mechanical, and viscoelastic properties are required to establish broader generalization.

The present validation addresses material-wise extrapolation only, while all experiments were conducted in water under a constant-temperature condition. Temperature can affect cavitation through changes in vapor pressure, liquid viscosity, surface tension, and dissolved-gas behavior, and hydrodynamic cavitation studies have demonstrated substantial temperature-dependent changes in cavitation intensity and flow regime [37]. Although such cloud-cavitation behavior cannot be directly transferred to the present single-bubble experiments, the constant-temperature assumption remains an important applicability limitation of the present model.

Temperature is also relevant to the material response because the dynamic mechanical and viscoelastic properties of polymers are temperature dependent. Consequently, temperature variations may influence the measured impact response through both changes in bubble dynamics and changes in the transient mechanical response of the polymer. Future studies should therefore systematically vary temperature and incorporate temperature, together with the corresponding temperature-dependent liquid and polymer properties, into the machine-learning feature space. The present experimental approach also has limitations in resolving the detailed flow and collapse mechanisms around the bubble. High-speed optical imaging provides information on the bubble geometry and collapse sequence, but does not directly quantify the surrounding liquid velocity field, microjet velocity, or the separate contributions of microjet impact and collapse-induced pressure waves. Future application of particle image velocimetry (PIV) could provide quantitative measurements of the liquid velocity and jet development, particularly under near-wall collapse conditions. Such measurements would be valuable for directly examining the physical mechanisms underlying the stand-off-dependent impact response identified in the present study. X-ray-based imaging techniques may provide complementary information for optically inaccessible or dense multi-bubble conditions, including internal bubble structures, void-fraction distributions, and bubble-cloud dynamics.

## 5. Conclusions

This study demonstrated that the impact load generated by laser-induced bubble collapse on polymer materials can be predicted using random forest regression. Experiments were conducted for polyethylene, PTFE, polyamide, and antistatic PET under different laser energies and stand-off distances, and the measured PVDF signals were converted into impact loads using material-specific calibration equations.

Among the tested regression models, random forest regression gave the best overall performance, achieving RMSE = 0.6820 N, MAE = 0.5339 N, $R^{2}=0.9168$, and MAPE = 12.44%. Its higher accuracy compared with linear regression, ridge regression, LASSO regression, and MLP regression indicates that the relationship between bubble-collapse impact load and the governing variables is strongly nonlinear.

The SHAP-based feature-importance analysis identified bubble collapse time, acoustic impedance, and loss modulus as the most influential predictors. These results indicate that the measured impact load is not governed by bubble dynamics alone, but by the combined effects of collapse timing, stress-wave transmission, and viscoelastic energy dissipation in the polymer target. The near-wall analysis at γ=1 further suggested that storage and loss moduli provide important predictive information under strongly confined collapse conditions.

Normalization by the equivalent acoustic impedance reduced part of the material-dependent variation in impact load, but residual differences remained among the polymers. This indicates that acoustic impedance is an important scaling parameter, but it is not sufficient as a universal descriptor of the impact response. Instead, the bubble-collapse impact load should be interpreted as a multivariable response involving bubble dynamics, acoustic coupling, viscoelastic behavior, and stand-off condition.

The learning-curve and leave-one-material-out validation results show that the present model is mainly applicable within the investigated experimental parameter space. Its ability to extrapolate to unseen polymer materials remains limited, and the model should not be interpreted as a direct predictor of cumulative cavitation erosion, fatigue life, or cloud-cavitation damage. Future work should include additional polymer materials, temperature-dependent liquid and material properties, and direct characterization of multi-bubble or cloud-cavitation behavior to improve model generalization and extend the framework toward practical hydraulic applications.

## Figures and Tables

**Table 1:** PVDF force calibration equations, voltage ranges, force ranges, and coefficients of determination for different materials.

Material	Voltage Range (V)	Force Range (N)	PVDF Force Calibration Equation	$R^{2}$
Polyethylene	0.020 to 0.062	1.98 to 3.79	F=44.94V+1.07	0.9678
PTFE	0.019 to 0.195	3.40 to 10.57	F=43.65V+2.06	0.9713
Polyamide	0.016 to 0.179	3.11 to 16.81	F=81.47V+2.34	0.9906
Antistatic PET	0.014 to 0.167	3.07 to 17.60	F=92.97V+2.44	0.9919

**Table 2:** Intrinsic material properties of the investigated polymers.

Material	Density (kg/m^3^)	Flexural Modulus (GPa)	Tensile Yield Strength (MPa)	Sound Speed (m/s)	Acoustic Impedance (MRayl)
Polyethylene	920	0.30	12	2088	0.57
PTFE	2200	0.50	20	1310	0.62
Polyamide	1150	2.60	51	2600	0.80
Antistatic PET	1340	3.29	105	2350	0.87

Density, flexural modulus, and tensile yield strength were obtained from engineering material databases [24]. The longitudinal wave velocity was compiled from published literature [25,26,27,28]. The acoustic impedance was calculated using Equation (6).

**Table 3:** Maximum bubble radius for different polymer materials, laser pulse energies, and normalized stand-off distances. Values are presented as mean ± standard deviation (n=3).

Laser Energy (mJ)	γ	Antistatic PET $R_{\max}$ (mm)	Polyamide $R_{\max}$ (mm)	PTFE $R_{\max}$ (mm)	Polyethylene $R_{\max}$ (mm)
12.5	1	0.595±0.145	0.530±0.130	0.545±0.115	0.530±0.115
	2	0.505±0.110	0.565±0.100	0.520±0.145	0.540±0.145
	3	0.565±0.135	0.545±0.120	0.525±0.140	0.510±0.140
	4	0.505±0.115	0.555±0.140	0.505±0.135	0.545±0.135
	5	0.520±0.125	0.505±0.110	0.535±0.150	0.515±0.150
25	1	1.085±0.130	1.010±0.120	1.050±0.140	1.030±0.140
	2	1.045±0.135	1.035±0.115	1.060±0.120	1.085±0.120
	3	1.045±0.115	0.985±0.115	0.970±0.145	0.995±0.145
	4	0.960±0.110	1.030±0.120	1.095±0.105	1.065±0.105
	5	1.015±0.115	1.025±0.130	1.025±0.110	1.045±0.110
50	1	1.385±0.115	1.355±0.135	1.350±0.115	1.380±0.115
	2	1.340±0.130	1.315±0.110	1.360±0.115	1.335±0.115
	3	1.310±0.110	1.260±0.140	1.300±0.110	1.320±0.110
	4	1.305±0.115	1.255±0.110	1.325±0.110	1.290±0.110
	5	1.370±0.135	1.305±0.115	1.385±0.120	1.355±0.120

$R_{\max}$ denotes the maximum equivalent bubble radius. Values are reported as mean ± standard deviation from three repeated measurements.

**Table 4:** Bubble collapse time for different polymer materials, laser pulse energies, and normalized stand-off distances. Values are presented as mean ± standard deviation (n=3).

Laser Energy (mJ)	γ	Antistatic PET $t_{c}$ (μs)	Polyamide $t_{c}$ (μs)	PTFE $t_{c}$ (μs)	Polyethylene $t_{c}$ (μs)
12.5	1	100.67±7.04	66.00±1.39	94.73±6.71	72.72±9.09
	2	88.47±6.52	81.20±7.71	96.60±6.63	60.60±5.25
	3	95.67±3.24	86.73±4.87	97.67±5.10	69.69±5.25
	4	88.07±1.50	92.00±10.11	92.07±0.99	72.72±9.09
	5	85.67±6.92	84.20±6.55	99.13±0.90	75.75±5.25
25	1	161.00±8.81	149.53±12.77	112.90±37.07	181.80±9.09
	2	171.73±16.97	147.60±5.44	160.30±6.27	169.68±5.25
	3	160.13±2.61	145.93±9.13	149.77±0.32	169.68±13.89
	4	155.20±3.20	147.47±6.59	162.67±25.75	166.65±5.25
	5	157.40±4.92	141.20±3.41	139.53±7.43	148.47±13.89
50	1	187.17±7.64	110.50±7.50	238.40±8.19	155.50±24.11
	2	228.83±5.77	170.50±2.50	220.53±3.21	206.33±3.82
	3	242.17±1.44	192.17±3.82	218.67±4.12	247.17±1.44
	4	247.17±1.44	193.00±2.50	235.03±33.64	250.50±4.33
	5	248.00±2.50	192.17±3.82	204.63±0.67	250.50±4.33

$t_{c}$ denotes the time interval from laser-induced plasma formation to the first complete bubble collapse. Values are reported as mean ± standard deviation from three repeated measurements.

**Table 5:** Input variables, symbols, units, and physical descriptions used in the experimental and predictive analyses.

No.	Variable	Symbol	Unit	Description
1	Distance to the solid surface	*h*	mm	Distance from the initial bubble center to the polymer surface. It is used to calculate the stand-off distance, $\gamma =h/R_{\max}$.
2	Maximum bubble radius	$R_{\max}$	mm	Largest bubble radius measured during the expansion stage from high-speed images.
3	Bubble collapse time	$t_{c}$	μs	Bubble collapse time measured using the PVDF sensor.
4	Laser pulse energy	$E_{L}$	mJ	Energy delivered by one laser pulse to generate the cavitation bubble.
5	Speed of sound in the solid	$c_{s}$	$m\;s^{-1}$	Velocity of a longitudinal stress wave through the polymer.
6	Combined acoustic impedance	*Z*	MRayl	Effective acoustic impedance at the water–polymer interface.
7	Flexural modulus	$E_{f}$	GPa	Material stiffness under bending deformation.
8	Storage modulus	$E^{\prime }$	GPa	Elastic part of the complex modulus, related to stored mechanical energy.
9	Loss modulus	$E^{\prime \prime }$	GPa	Viscous part of the complex modulus, related to dissipated mechanical energy.
10	Loss factor	tanδ	–	Ratio of loss modulus to storage modulus, $\tan\delta =E^{\prime \prime }/E^{\prime }$.
11	Relaxation time	τ	s	Time needed for viscoelastic stress to decrease after deformation.
12	Yield strength	$\sigma_{y}$	MPa	Stress where the polymer begins permanent plastic deformation.
13	Solid density	$\rho_{s}$	$\mathrm{kg}\;m^{-3}$	Mass of the polymer per unit volume, used for wave speed and impedance.

The symbol “–” indicates a dimensionless quantity. One MRayl is equal to $10^{6}\;\mathrm{kg}\;m^{-2}\;s^{-1}$, or equivalently $10^{6}\;\mathrm{Pa}\;s\;m^{-1}$.

**Table 6:** Bayesian optimization settings used for machine learning model comparison.

Aspect	Description
Optimization library	Optuna 4.9.0
Bayesian optimizer/sampler	Tree-structured Parzen Estimator (TPESampler)
Surrogate model type	TPE density estimator using l(x) and g(x) nonparametric likelihood models
Acquisition/candidate criterion	Expected-improvement-style TPE candidate selection using the l(x)/g(x) density ratio
Objective function	Minimize mean grouped 5-fold validation RMSE
Cross-validation design	Five condition-based folds grouped by material, normalized stand-off distance (γ), and laser pulse energy
Random seed	42
Startup random trials	8
EI candidates per trial	24
Bayesian optimization iterations	MLP Regressor = 35; Random Forest = 35; Ridge Regression = 25; Lasso Regression = 25

**Table 7:** Bayesian hyperparameter search space for each regression model.

No.	Method	Bayesian Hyperparameter Search Space
1	Random forest regression	n_estimators∈[100,800]; max_features∈[0.30,1.00]; max_depth∈{None,3,5,8,12,16,24}; min_samples_split∈[2,10]; min_samples_leaf∈[1,8]; bootstrap∈{true,false}; max_samples∈[0.60,1.00] when bootstrap=true
2	MLP regressor	hidden_layer_sizes∈{(16),(32),(64),(32,16),(64,32),(64,32,16)}; activation∈{relu,tanh}; $\mathrm{alpha}\in [10^{-5},10^{-1}]$ on a logarithmic scale; $\mathrm{learning}\_\mathrm{rate}\_\mathrm{init}\in [10^{-4},10^{-2}]$ on a logarithmic scale; learning_rate∈{constant,adaptive}; batch_size∈{auto,16,32,64}
3	Lasso regression	$\mathrm{alpha}\in [10^{-5},1]$ on a logarithmic scale; fit_intercept∈{true,false}; selection∈{cyclic,random}
4	Ridge regression	$\mathrm{alpha}\in [10^{-4},10^{3}]$ on a logarithmic scale; fit_intercept∈{true,false}

Linear regression was evaluated as an ordinary least-squares baseline and was therefore not included in the Bayesian hyperparameter search space.

**Table 8:** Bayesian-optimized hyperparameters of the regression models using all 13 input variables.

Method	Bayesian-Optimized Hyperparameters
Random forest regression	bootstrap=true, max_depth=16, max_features=0.8562, max_samples=0.7739, min_samples_leaf=1, min_samples_split=4, n_estimators=392
MLP regressor	activation=relu, alpha=0.0007015, batch_size=32, hidden_layer_sizes=(64), learning_rate=adaptive, learning_rate_init=0.002768, solver=adam
Lasso regression	$\mathrm{alpha}=1.0147\times 10^{-5}$, fit_intercept=true, selection=cyclic
Ridge regression	alpha=0.0002946, fit_intercept=true

**Table 9:** Prediction performance of the regression models using all 13 input variables.

Method	RMSE	MAE	$R^{2}$	MAPE (%)
Random forest regression	0.6820	0.5339	0.9168	12.44
MLP regressor	0.7183	0.5651	0.9127	13.66
Ridge regression	1.0713	0.8461	0.7994	20.44
Lasso regression	1.0715	0.8455	0.7995	20.37
Linear regression	1.0725	0.8444	0.7989	20.31

**Table 10:** Comparison of cross-validation methods using the optimized Random Forest model.

Validation Method	RMSE	MAE	$R^{2}$	MAPE (%)
Grouped 5-fold CV	0.6820	0.5339	0.9168	12.44
Repeated grouped 5-fold CV	0.7518	0.5624	0.9200	13.59
Leave-one-condition-out CV	0.6720	0.5079	0.9361	11.88

**Table 11:** Feature ranking based on mean absolute SHAP values from the random forest regression model.

Rank	Feature	Mean Absolute SHAP Value
1	Bubble collapse time, $t_{c}$	1.406
2	Acoustic impedance, *Z*	1.050
3	Loss modulus, $E^{\prime \prime }$	0.549
4	Storage modulus, $E^{\prime }$	0.390
5	Tensile yield strength	0.240
6	Solid density	0.223
7	Bubble radius, *R*	0.215
8	Distance to solid, *h*	0.170
9	Sound speed in the solid	0.151
10	Flexural modulus	0.124
11	Laser energy	0.114
12	Effective relaxation time, $\tau_{\mathrm{eff}}$	0.043
13	Loss factor, tanδ	0.010

## Data Availability

The original contributions presented in this study are included in the article. Further inquiries can be directed to the corresponding author.

## Abbreviations

The following abbreviations are used in this manuscript:

APET	Antistatic polyethylene terephthalate
DMA	Dynamic mechanical analysis
fps	Frames per second
LASSO	Least Absolute Shrinkage and Selection Operator
LED	Light-emitting diode
MAE	Mean absolute error
MAPE	Mean absolute percentage error
ML	Machine learning
MLP	Multilayer perceptron
Nd:YAG	Neodymium-doped yttrium aluminum garnet
PA	Polyamide
PA66	Polyamide 66
PE	Polyethylene
PET	Polyethylene terephthalate
PTFE	Polytetrafluoroethylene
PVDF	Polyvinylidene fluoride
RF	Random forest
RMSE	Root mean squared error
SHAP	Shapley additive explanations
